# Host species and geography impact bee-associated RNA virus communities with evidence for isolation by distance in viral populations

**DOI:** 10.1093/ismeco/ycad003

**Published:** 2024-01-10

**Authors:** Chris R P Robinson, Adam G Dolezal, Irene L G Newton

**Affiliations:** Department of Biology, Indiana University, Bloomington, IN 47405, United States; Department of Entomology, University of Illinois Urbana-Champaign, Urbana, IL 61801, United States; Department of Biology, Indiana University, Bloomington, IN 47405, United States

**Keywords:** RNA viral ecology, metatranscriptomics, isolation-by-distance, host-associated symbionts

## Abstract

Virus symbionts are important mediators of ecosystem function, yet we know little of their diversity and ecology in natural populations. The alarming decline of pollinating insects in many regions of the globe, especially the European honey bee, *Apis mellifera*, has been driven in part by worldwide transmission of virus pathogens. Previous work has examined the transmission of known honey bee virus pathogens to wild bee populations, but only a handful of studies have investigated the native viromes associated with wild bees, limiting epidemiological predictors associated with viral pathogenesis. Further, variation among different bee species might have important consequences in the acquisition and maintenance of bee-associated virome diversity. We utilized comparative metatranscriptomics to develop a baseline description of the RNA viromes associated with wild bee pollinators and to document viral diversity, community composition, and structure. Our sampling includes five wild-caught, native bee species that vary in social behavior as well as managed honey bees. We describe 26 putatively new RNA virus species based on RNA-dependent RNA polymerase phylogeny and show that each sampled bee species was associated with a specific virus community composition, even among sympatric populations of distinct host species. From 17 samples of a single host species, we recovered a single virus species despite over 600 km of distance between host populations and found strong evidence for isolation by distance in associated viral populations. Our work adds to the small number of studies examining viral prevalence and community composition in wild bees.

## Introduction

While long known to be important to human and agricultural health, viral symbionts are increasingly considered as critical modulators of ecosystem function [1, 2]. Despite their importance, virus ecology in natural populations of host species remains understudied. Although viral symbionts are all in some way parasitic and are best characterized by the detrimental fitness effects on their host, all host–virus interactions occur along a continuum where the cost of viral association may be nearly neutral. The co-evolving dynamics of virus–host interactions, shaped by myriad factors such as transmission rate, host density, or pathogen virulence, can be perturbed by minor changes in host ecology, resulting in rapid changes in host–virus dynamics and antagonistic changes such as increases in virus transmission, virulence, and spread as evidenced by recent virus pandemics. Efforts to understand the impact of pathogen burden on ecosystems function, such as in the conservation of threatened species, necessitate a deeper understanding of virus ecology.

Among the most alarming population declines are those of pollinating insects, which provide essential ecosystem and agricultural services across many environments [3]. For most of the world, the European honey bee *Apis mellifera* is the key managed pollinator, but wild bees have been shown to be important contributors to crop yield and agricultural function [4], as well as in their native ecosystems [3]. The high number of colony losses from both native and nonnative bee populations in some parts of the world [5–7] has been driven by a confluence of factors such as habitat loss, pesticide use, and pathogen exposure [8]. Among pathogens, the emergence of new or more widespread virus infections have garnered the most attention. In bees alone, there are over 50 described viruses [9, 10], many of which have important implications for host survival [6, 11]. However, despite the widespread and urgent concern about the role viruses play in the decline of bee populations worldwide, it remains that the majority of bee viral research has been conducted only in *A. mellifera*. Our understanding of virus ecology in other bee species, which often share overlapping pollinator networks with *A. mellifera*, is far more limited [12, 13]. A number of studies have examined the transmission of honey bee viruses to other bee species [6, 14–16], identifying the evidence of virus pathogen transmission vectored by *A. mellifera*. Although these studies have been important in describing the effects of virus infection and in illuminating pathogen networks among pollinating bees, they have focused almost exclusively on the viruses known to infect honey bees and have excluded investigating the native viromes of wild bee species. The absence of these data, addressed by only a handful of studies [10, 17] precludes the development of a baseline understanding of virus ecology in wild bee species and limits the predictors of virus diseases in wild bees to the presence of sympatric honey bee populations.

Due to the many interactions intrinsic to pollinator networks, there are many potential opportunities for intra- and interspecific pathogen transmission, including the consumption of common resource stocks [18], vector-mediated transmission [19–21], and through sharing of the same floral resources [14, 22, 23]. Given the number of direct and indirect interactions between communities of pollinating bees, it is difficult to anticipate the degree of genetic and taxonomic diversity within bee-associated virus communities. Nearly all insect-infecting viruses are RNA viruses, which are generally characterized by relatively high mutation rates resulting from their lack of effective proofreading activity in their RNA polymerase [9]. This error-prone nature of RNA viruses can lead to high amounts of virus genetic diversity within a given host [9]. The fate of these genetic variants is then determined by ecological and evolutionary forces, such as selection, drift, and migration. Bee-associated RNA virus populations, undergoing weak to neutral selection, would be expected to be associated with high degrees of genetic diversity within the population. Low genetic diversity associated with RNA virus populations might be expected from positive selection on virus variants, such as from immune system responses to infection or arising from demographic changes, such as population bottlenecks originating from recent transmission events.

Inter- and intraspecific transmission of RNA virus population is expected to be a major source of virus infection among pollinating bees [22], and social behavior of bee species might have a significant effect on the genetic and taxonomic variation of associated virus communities due to the increased opportunity for migration between viral populations. Species of pollinating bees can differ tremendously in behavior and the degree of sociality. Nearly all bees provide resources to the next generation of offspring, though how these resources are provided and distributed vary widely. Solitary bee offspring may receive only a single provisioning of resources to complete their development into adults [24], whereas eusocial bees, such as *A. mellifera*, routinely share food between individuals and between developing offspring and might result in many more opportunities for the transmission of virus symbionts. Investigations of the bacterial communities of solitary bees as well as social bees reveal that the communities are often highly host-specific and consistent between individuals [25, 26]. Within other species of Hymenoptera, including the ants, the host-specificity of the associated bacterial community seems to be more relaxed [27].

Here, we conducted metatranscriptomic sequencing of five species of solitary and social halictid bees as well as the highly eusocial *A. mellifera*. The major goal of this study was to compare the diversity, abundance, and structure of RNA viral communities associated with five species of wild bee pollinators in order to better understand the ecology and distribution of RNA viruses associated with these host bee species. We chose to examine halictid bee species because of their wide geographic distribution and because of their importance as generalist pollinators, especially *Lasioglossum leucozonium*. Further, sociality has evolved and been lost repeatedly within this group, resulting in a phylogeny of many closely related species that demonstrate a spectrum of social behavior [25, 28–30]. Consequently, our sampling includes both solitary and social *Halictidae* species. Samples from sympatric *A. mellifera* populations were included as a comparison since it represents a phylogentically distance eusocial species and is the most deeply studied bee species. Our sampling included five bee species from a single location and a single bee species distributed across five sites in the Northeastern USA For each sample, we sought to (i) characterize the RNA viruses associated and describe novel viruses associated with the bee host, (ii) explore whether host-associated factors such as species or behavior impact RNA viral community diversity or composition, (iii) identify whether known honey bee viruses are present in the RNA viral communities associated with the sampled host. With these approaches, we document the viral communities associated with multiple sympatric host species and provide evidence for isolation by distance of RNA viral metapopulations associated with a solitary host species.

## Methods

### Sample collection

**Figure 1:**
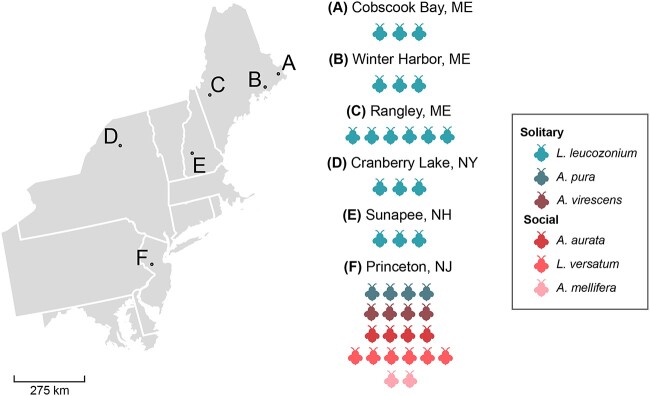
Locations of sample sites and species nomenclature; letters on map correspond to individual sampling sites; the name of each sampling site and the number of bees collected at each site is to the right of the map; the total number of bees icons associated with a given sampling site represents the number of bee samples collected at the site.

Individual bee samples were collected from the Mid-Atlantic and Northeastern USA in 2016 and 2018 (Fig. 1). A total of 38 samples were collected across six species of bee. Two samples were removed from further analysis due to poor assembly. The species *Augochlora pura*, *A. mellifera*, *Agapostemon virescens*, *Lasioglossum versatum*, and *Augochlorella aurata* were collected from Princeton, New Jersey. *L. leucozonium* was collected from several sites in the Northeastern US. These sites were Cobscook Bay, ME (44.839159, -67.150384); Winter Harbor, ME (44.394613, -68.084561); Rangley, ME (44.928539, -70.636231); Craneberry Lake, NY (44.204063, -74.831175); and Sunapee, NH (43.382356, -72.085406) (Supplementary Table S1).

### RNA library preparation and sequencing

RNA libraries were constructed from each individual sampled bee. The entire body of the bee was used for RNA extraction except for *A. mellifera* in which only the abdomen was used for extraction. In brief, individual bee samples were homogenized with sterile plastic pestles and treated with proteinase K. RNA was extracted using Zymo Quick-RNA Miniprep Plus kits. Host rRNA was removed using the NEBNext rRNA Depletion Kit (NEB #6310). Individual RNA libraries were created for each bee sample with NEBNext Ultra II Directional RNA Library Prep Kits. Each single-end 100 bp RNA library was sequenced on an Illumina NovaSeq 6000 at Princeton University across three flowcells.

### RNA virus characterization, annotation, and discovery

Raw reads were quality checked with FastQC [31] and MultiQC [32] before trimming with Trimmomatic [33]. HiSat2 [34] was used to align reads from each individual RNA library to a corresponding reference genome. At the time of analysis, *L. versatum* did not have a reference genome, and reads from these samples were aligned to the *L. leucozonium* reference genome. Except for *A. melifera*, reference genomes for all bee species were generated by the labs of Sarah Kocher and Erez Lieberman Aiden [35–37]. RNA reads that aligned to the reference were discarded, and the unaligned reads were used as input for assembly with rnavirusSPADES [38]. Assembled contigs were then used as inputs for CheckV [39] and Cenote-Taker2 [40] to assign initial confidence scores that a given contig was virus. We culled contigs that were either <500 base pairs or in which the presence of a virus hallmark gene could be confidently called. Viruses that passed this QC and maintained a sufficient virality score were annotated with Cenote-Taker2. We considered viral genomes nearly complete if they were considered greater than 85% complete by CheckV [39].

Virus contigs were then screened for partial or complete RNA-dependent RNA polymerase (RdRP) sequences using annotations from Cenote-Taker 2 and Palmscan [41]. RdRP sequences were considered truly RdRP-associated if they were identified by both Cenote-Taker 2 and Palmscan or if Palmscan associated the sequence with a high confidence score. Virus contigs containing a partial or complete RdRP sequence were then translated and clustered into family-equivalent operational taxonomic units (OTUs) using CD-HIT [42]. If two or more RdRP translations shared *>*40% amino acid identity (AAI) over 80% of the sequence length, they were clustered into the same family-equivalent OTU. Contigs from each cluster were then queried with BLASTP [43] to assign putative virus taxonomy. A virus species was considered novel if it shared *<*90% RdRP AAI across 80% of the sequence length with known viral RdRPs in the nonredundant NCBI database [44].

### Phylogenetic analysis of RNA viruses

The RdRP gene in RNA viruses is the standard in taxonomic comparisons between family-equivalent OTUs of RNA viruses due to the conserved nature of its sequence relative to the sequence of other genes in RNA virus genomes [45]. To place the putatively novel RdRP sequences from our dataset within existing viral phylogenies, all known genomes associated with each virus family identified in this study were downloaded from NCBI RefSeq. Viral families were selected on the basis that they were associated with a putatively novel RdRP found in this study. Palmscan and custom bash scripts were used to identify and extract RdRP sequences in the NCBI virus genomes. RdRP sequences specific to each virus family, either from NCBI or from samples in this study, were aligned with MAFFT [46] and trimmed with TrimAL with the *-automatic1* flag [47].

This alignment was then used to estimate maximum likelihood trees with IQTREE2 [48] with 1000 bootstrap replicates using the most appropriate model found by ModelFinder [49]. A combination of bootstrap scores and SH-like branch support was used to validate the tree topology of nodes in the estimated tree. In all trees, the *Astroviridae* virus, *Mamastrovirus 3*, was used as an outgroup. The result of ModelFinder, the associated AIC and BIC scores, and the number of amino acids sites for each viral family are provided as a Supplemental Table (Supplementary Table S2).

### RNA virus diversity and abundance

Absolute and relative virus abundances were estimated in each bee sample by mapping non-bee raw reads with bwa-mem [50] to each contig identified as virus. The number of mapped reads to a given contig was then normalized by the total number of reads in the sequencing library and the overall length of the contig as suggested by Roux *et al*. [51].

The alpha diversity metrics, Shannon diversity, Simpson diversity, and richness were calculated among the samples at the species and family level using the Rhea script sets [52]. Comparing differences among samples separated by species or social behavior was done using a Tukey’s Honest Significant Difference Test with the R package *agricolae* [53]. We calculated beta diversity by quantifying virus diversity structure as composite variables along two axes of a principal coordinate ordination analysis and tested it using Adonis tests from the vegan [54] package. These analyses were performed using R 4.2.2 and plotted with ggplot2 [55].

### F_st_ calculations and analysis

To investigate the variation in RNA virus populations associated with allopatric populations of *L. leucozonium*, the cluster of contigs associated with *L. leucozonium* and from the family *Narnaviridae* was mapped with BWA-mem against the longest length contig within the cluster. Variant calling was done using LoFreq [56] without Indel calling. Variant calling files produced by LoFreq were used to calculate Nei’s *G_st_* (*F_st_*) (Equation (1)) for each virus subpopulation (e.g. single sample of bee). We built a custom Python script to calculate *F_st_* (https://github.com/en-nui/BeeSocialityMetatranscriptomics).


$$ {G}_{s\mathrm{t}}=\frac{\left({H}_{\mathrm{Total}}-{H}_{\mathrm{Within}}\right)}{H_{\mathrm{Total}}} $$

$$ {H}_{\mathrm{Total}}=\frac{N_{\mathrm{Total}}}{N_{\mathrm{Total}}-1}\left(1-\sum_{i=1}^Np{-}_i^2\right) $$

$$ {H}_{\mathrm{Within}}=\frac{N_{\mathrm{Subpopulation}}}{N_{\mathrm{Subpopulation}}-1}\left(1-\sum_{i=1}^N{p}_i^2\right) $$

**Equation 1.** Calculations for Nei’s *G_st_* where the difference in average heterozygosity of the total population *H_Total_* and a given subpopulation *H_Within_* is normalized by the average heterozygosity of the total population. *NT_otal_* and *N_Subpopulation_* are the total number of sampled chromosomes across all populations and within one subpopulation, respectively. *P_i_* is the frequency of allele *i*.

*F_st_* was calculated globally among all 17 *Narnaviridae* populations. Additionally, we calculated pairwise *F_st_* for each of the virus populations. Finally, each *Narnaviridae* population that shared a common sampling site (e.g. virus populations associated with a host that was sampled from the same sampling site) was grouped together as a single population, and we repeated the *F_st_* calculation for each of the geographically defined virus populations. *F_st_* values in both analyses were then compared across a distance matrix, where pairwise Haversine distances (km) between sampling sites were compared to the corresponding differences in allele frequency variation using a Mantel test with the Pearson correlation method from the Vegan package.

## Results

### Characterization of known and novel viruses from bee hosts

We sequenced the RNA virus communities of 20 *Apidae* and *Halictidae* bees collected from a single site (Princeton, NJ) and an additional 18 bees from a single *Halictidae* species were sampled from five sites in the Northeastern USA (Fig. 1). Sequencing produced 1 574 309 491 reads and, after quality filtering, individual libraries specific to each species were mapped against corresponding host genomes and assembled into 156 912 contigs. We chose a conservative approach to minimize false discovery and relied on stringent filtering of contigs by length and for known virus hallmark genes, reducing the total number of true virus contigs to 110. From these contigs, we characterized a total of 26 putatively novel virus species OTUs from 12 different virus families based on RdRP sequence similarity to known viral RdRP sequences. The majority of the viral contigs contained mostly complete virus genomes and included a known RdRP or capsid gene.

**Figure 2:**
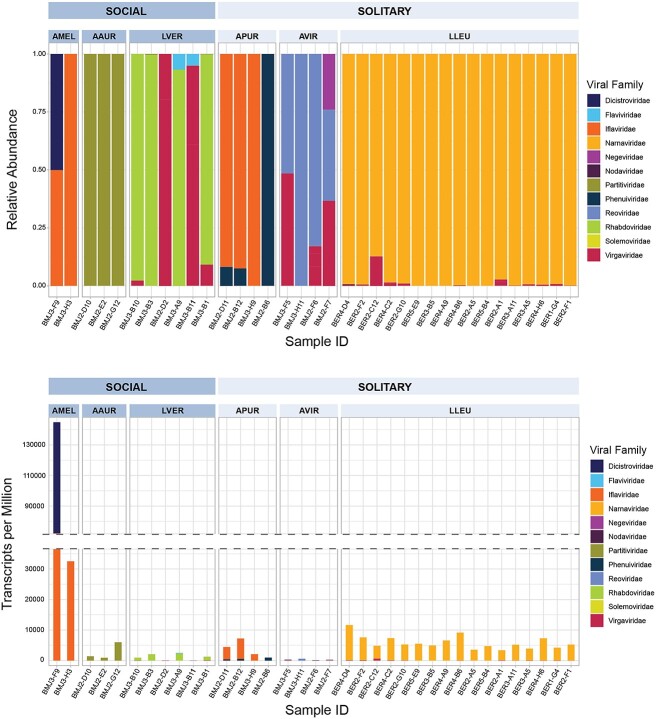
Relative abundance (A; top) and absolute abundance (B; bottom) of normalized RdRP virus transcripts across sampled bees in this study; each bar plot along the *x*-axis represents an individual sampled bee, and the *y*-axis represents the proportion (top) or absolute number (bottom) of virus reads assigned to each viral family; a dotted horizontal line in the bottom panel (B) is used to demarcate the higher viral loads in honey bee hosts.

A virus species was considered novel if it shared *<*90% RdRP AAI across 80% of the sequence length with known viral RdRPs in the nonredundant NCBI database. Clustering of virus OTUs was guided by recent comparisons of inter- and intrapopulation variation in virus populations [57] and by recommendations of the International Committee on Taxonomy of Viruses [58]. Virus species were clustered by *>*95% shared AAI over 80% of the sequence length. Virus families were clustered by *>*40% AAI. BLASTP of all translated RdRP sequences resolved the species identity of 3 out of 29 species OTUs. The remaining 26 species OTUs, distributed among all 6 sampled bee species, were resolved only to the family level and are considered to be putatively novel virus species. Across all 38 sampled bee hosts, we recovered 8 near-complete viral genomes, 6 of which were associated with putatively novel viral species. We considered viral genomes nearly complete if they were considered greater than 85% complete by CheckV [39]. Families representing important honey bee virus pathogens, such as *Dicistroviridae* and *Iflaviridae*, were found in our survey with *Iflaviridae* appearing in both *A. mellifera* and the solitary ground nesting bee, *A. pura* (Fig. 2A and B). Among halictid hosts, the abundance of viruses (detected by depth of read mapping to contigs) varies between host species, with *A. pura* and *L. versatum* maintaining the highest levels of virus transcripts compared to *Augochlora aurata* and *A. virescens*. The virome of *A. virescens* was dominated equally by *Reoviridae* and *Virgaviridae* at levels much lower than halictids sampled in this study, and this may be a result of virus-infected plant tissue associated with pollen or plant debris consumed by the bee prior to RNA extraction.

Halictid virus transcripts were relatively low compared to extremely high levels of virus transcript in *A. mellifera* samples (Fig. 2B, Supplementary Fig. S5). *A. mellifera* is known to persistently harbor viruses from *Iflaviridae* and *Dicistroviridae* [6]. Almost all bee-associated virus families characterized in this survey are known to infect insects, though we identified several virus species related to known plant pathogenic viruses. In addition, a single species from the family *Narnaviridae*, uncommon outside of fungal hosts, dominates the virome of all sampled *L. leucozonium*.

### Novel RNA viruses

**Figure 3:**
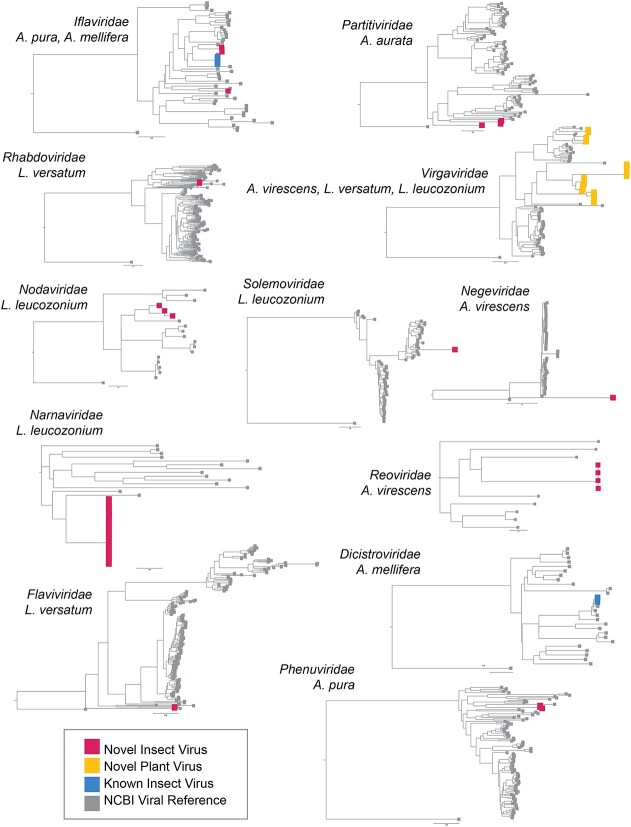
Overview of viruses found in bees sampled in this study; bee hosts associated with a virus family are listed underneath the corresponding family name (*Rhabdoviridae* excluded); viruses associated with this study are separated into three groups; novel insect-associated viruses, known insect-associated viruses, and viral genomes taken from NCBI RefSeq; described phylogenies were generated from RdRP gene sequence alignments.

**Figure 4:**
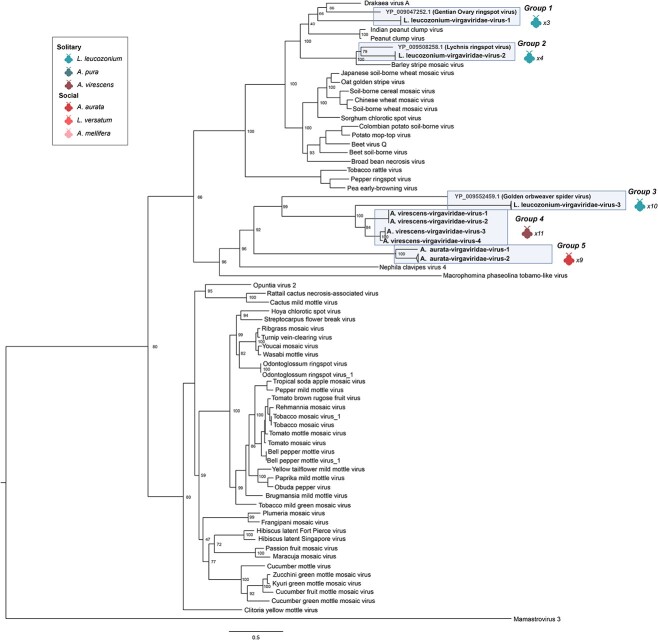
RdRP phylogeny of *Virgaviridae* viruses; tree is midpoint rooted for clarity only. Viruses sampled in this study are highlighted in blue; bootstrap values from 1000 replicates for select nodes are provided; bee icons represent species host that virus group was associated with; numbers represent the number of bee samples associated with virus group; the scale bar represents the number of amino acid substitutions.

We identified novel species in 11 of the 12 virus families associated with sampled bees in this study (Fig. 3, Supplemental Figures 7–15). The majority of novel virus species fell into the family *Virgaviridae* which is primarily composed of plant pathogenic viruses (e.g. tobacco mosaic virus). *Virgaviridae* species accounted for 9 of the 26 (35%) of the novel RNA viruses identified in this study and were recovered from *A. virescens*, *L. versatum*, and *L. leucozonium*. Individual *L. leucozonium* samples harbored high *Virgaviridae* diversity and were associated with six of the nine novel *Virgaviridae* species. The first of these species (Fig. 4, Group 1) is most closely associated with gentian ovary ringspot virus (YP 009047252.1), a virus first isolated in 2014 and is vectored by pollinating insects [59]. Long branch lengths are indicative of high levels of divergence, although clustering by AAI of known and novel virus RdRPs places this clade into the genus *Goravirus*. The second *Virgaviridae* species (Fig. 4, Group 2) identified in *L. leucozonium* shares the highest identity with Lychnis ringspot virus (YP 009508258.1), a plant pathogen of dicot plants. Amino acid clustering of all RdRPs in this clade falls into the genus *Hordeivirus*, which is known to be transmitted by both pollen and seed [60]. The remaining six novel species (Fig. 4, Groups 3–5) share high sequence divergence with one another and with the closest known relative, *Nephilia clavipes virus 3* (YP 009552459.1), recovered from the Golden Orb-Weaver spider [61]. *Virgaviridae* transcripts were uniformly low in all isolates, and it is difficult to distinguish whether the association of these virus species is due to infection of bee body issue, or if they were associated with plant debris on or consumed by bees during foraging. In the case of this study, the nine novel *Virgaviridae* species are most likely associated with the pollen foraged or consumed directly by these three bee species and reflect the significance of pollinators as vectors of plant pathogenic viruses.

Each of the five halictid species harbored novel virus species that were unique to each halictid species host. The viromes of the social halictid *A. aurata* were dominated by a single species of *Partitiviridae*, (Supplementary Fig. S13, Clade 1). RdRP sequences within this species were most closely related to *Cryptosporidium pavum* 1 virus (YP 009508065.1) [62], though external long branches of this clade suggest significant sequence divergence. Likewise, the other social halictid sampled in this survey, *L. versatum*, was associated with *Virgaviridae* and a single *Rhabdoviridae* and *Flaviviridae* species (S15). The number of assigned *Rhabdoviridae* transcripts in these samples was much higher than the number of transcripts assigned to *Virgaviridae*, suggesting that *Rhabdoviridae* association in *L. versatum* reflects an active virus infection (Supplementary Fig. S12). The solitary halictids harbored a higher absolute number of virus species compared to the social halictids, though this difference in diversity was not statistically significant. We identified two novel species of *Iflaviridae* (discussed further in the text) and two species of *Phenuiviridae* across all five samples. This species of *Phenuiviridae* was most similar to the *Mothra mobuvirus* (YP 009666266.1) (Supplementary Fig. S7), and RdRP sequence clustering places both viruses within the genus *Mobuvirus*. The final solitary halictid sampled from Princeton, *A. virescens*, harbored four novel species of virus across *Negeviridiae* (Supplementary Fig. S8), *Reoviridae* (Supplementary Fig. S9), and *Virgaviridae*. RdRP sequences in these samples were highly divergent and showed no close association with any known ssRNA viruses in NCBI RefSeq (Fig. 3). Virus transcripts in these samples were incredibly low and were similar to other *Virgaviridae* transcript levels in other sampled bee isolates, suggesting that these viruses are passively associated with environmental debris associated with or consumed by *A. virescens* during sampling.

**Figure 5:**
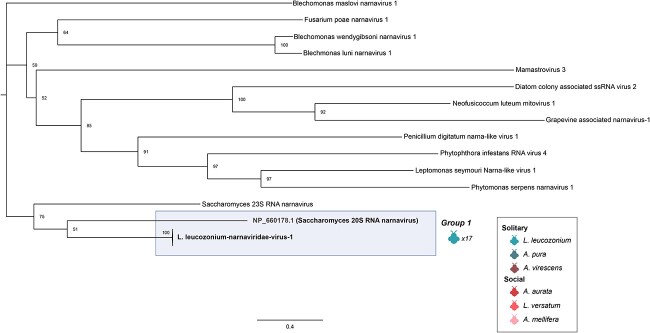
RdRP phylogeny of *Narnaviridae* viruses; tree is midpoint rooted for clarity only; viruses sampled in this study are detailed highlighted in blue; bootstrap values from 1000 replicates for select nodes are provided; bee icons represent species host that virus group was associated with; numbers represent the number of bee samples associated with virus group; the scale bar represents the number of amino acid substitutions.

The solitary halictid, *L. leucozonium*, sampled across five allopatric populations showed a surprising trend. As discussed above, individual samples of *L. leucozonium* were associated with a high diversity of *Virgaviridae* species, possibly reflecting a diversity of pollen samples consumed by this species during foraging. In contrast to this diversity of plant pathogenic viruses, all 17 samples of *L. leucozonium* were infected by a single species of *Narnaviridae*. This species is most closely related to *Saccharomyces 20S* RNA narnavirus (NP 660178.1) (Fig. 5).

### No evidence of known honey bee viruses in wild bee hosts

**Figure 6:**
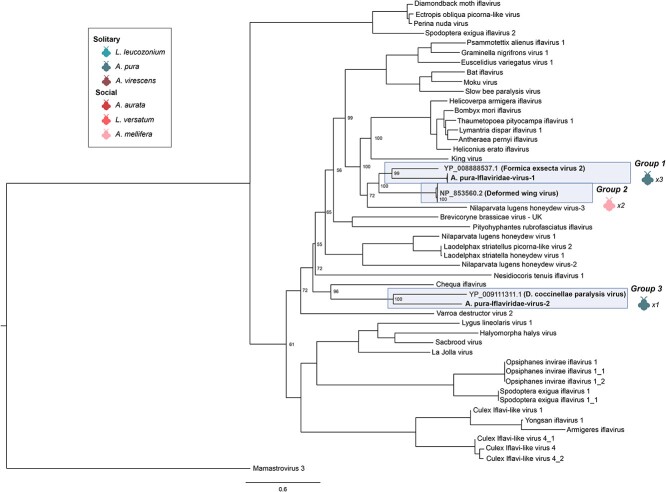
RdRP phylogeny of *Iflaviridae* viruses; tree is midpoint rooted for clarity only; viruses sampled in this study are highlighted in blue; bootstrap values from 1000 replicates for select nodes are provided; bee icons represent species host that virus group was associated with; numbers represent the number of bee samples associated with virus group; the scale bar represents the number of amino acid substitutions.

Species-level OTU clustering of RdRP protein sequences revealed three virus species distributed across *A. mellifera* and *A. pura* (Fig. 6). Though *Iflaviridae* have been shown to widespread virus symbionts of insects [63], previous work has shown that viruses in this family, particularly deformed wing virus, are a major cause of the worldwide decline in honey bee populations [64-66] and are important pathogens of other pollinators such as bumblebees [67]. BLASTP of the RdRP protein sequence identified that deformed wing virus, one of the three species of *Iflaviridae* (Fig. 6, Group 2) found in our survey, was found in both of the honey bee samples, and combined with the extremely high level of virus transcript found in these samples (Fig. 2B), it is indicative of active virus infection—possibly a result of feeding by the parasitic mite, *Varroa destructor* [66].

Further, we identified two novel virus species in *Iflaviridae*. Phylogenetic analysis of shared virus RdRPs in *Iflaviridae* revealed that a virus species infecting *A. pura* (*A. pura*–*Iflaviridae*-1 virus through *A. pura*–*Iflaviridae*-2 virus) is most closely related to *Formica exsecta virus 2* (Fig. 5, Group 1), a virus associated with the ant *F. exsecta* [68]. Long branch lengths separating the *F. exsecta* 2 virus and *A. pura*–*Iflaviridae*-1 virus suggest relatively long periods of divergence. Members of the clade occupied by deformed wing virus formed the next most related group. The final novel *Iflaviridae* virus species, *A. pura*–*Iflaviridae*-2 virus, was found to be most similar to the virus *Dinocampus coccinellae* paralysis virus (Fig. 5, Group 3). This virus has been shown to induce behavior manipulation, such as tremors and paralysis, of ladybird beetles that have been parasitized on by the wasp, *Dinocampus coccinellae* [69]. Similar to above, long branch lengths between this virus and *A. pura–Iflaviridae*-2 virus indicate high levels of divergence.

### Evidence for isolation by distance in wild bee-associated viral populations

*L. leucozonium* hosts sampled from all five sample sites were found to be associated with viruses from the family *Narnaviridae*. Despite over 600 km of distance between some sites and the low replication fidelity intrinsic to RNA viruses, RNA virus populations in these samples are remarkably similar (Fig. 5). Each sample of *L. leucozonium* is dominated by a putatively novel virus in the virus family *Narnaviridae*. Membership in this family was assigned by comparing the translated nucleotide identity (AAI) of the virus gene encoding RdRP. RdRP sequences from all *L. leucozonium*-associated *Narnaviridae* shared 95% AAI over 80% of the sequence length, indicating that *Narnaviridae* found in each *L. leucozonium* belongs to the same species (Fig. 5). Viruses within *Narnaviridae* have positive-sense single-stranded RNA genomes of 2.3–2.9 kb [70] and encode a singular RdRP protein. We recovered a complete *Narnaviridae* genome from each individual *L. leucozonium* with an average length of ~3.2 kb. Sequencing metrics for *Narnaviridae* in *L. leucozonium*, including relative read abundance and genome-wide coverage, are included as a Supplemental Table (Supplementary Table S3). Absolute abundances of normalized viral transcripts in *L. leucozonium* alongside the average normalized viral transcript across all sampled halictid hosts, as well as abundances for individual viral species, are provided as a set of Supplemental Figures (Supplementary Fig. S16, Supplementary Figure S17–28).

The presence of the same virus species among all sampled *L. leucozonium* individuals allowed us to compare the variation in allele frequency of subpopulations of *Narnaviridae* (e.g. one sampled bee host) to the variation in allele frequency of the entire population of RNA viruses (e.g. between all sampled bee hosts across all sample sites). To explore this variation, we developed a custom Python script to calculate Nei’s Gst [71] for pairwise *F_st_* comparisons between each single *Narnaviridae* population and *Narnaviridae* populations from each sampling site. We calculated a global *F_st_* of 0.484, which is indicative of high interpopulation genetic variation. Results from the individual pairwise *F_st_* calculations produced *F_st_* values indicating high levels of population differentiation with an average *F_st_* of 0.322 (Supplementary Fig. S6, Supplementary Fig. S9). Though there was significant amount of differentiation between individual *Narnaviridae* populations, a Mantel test using Spearman’s rank correlation found no association with pairwise *F_st_* and Haversine distance (*P* = .607).

**Figure 7:**
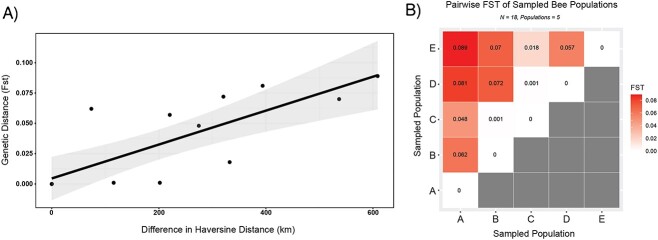
Scatterplot depicting pattern between pairwise Haversine distance (km) and F_st_ (A; left); individual points represent populations of *Narnaviridae* associated with bee hosts sampled from the same site; a Mantel test produced a significant correlation between the Haversine distance between any two sampling sites and pairwise F_st_ (*P* = .025); plotted linear regression is not representative of the Mantel test but is for clarity only; a matrix of pairwise F_st_ comparisons representing patterns of genetic differentiation among *Narnaviridae* populations associated with five geographically defined sites (B; right); letters on the *x*-axis correspond to sampled *L. leucozonium* population (Fig. 1 ; shading represents the degree of genetic differentiation between any pair of combinations; darker hues indicates higher values of differentiation; genetic differentiation is defined by a scale on the right side of the figure.

Pairwise *F_st_* values produced by comparing *Narnaviridae* associated with geographically defined hosts showed a contrasting trend. Calculations of pairwise *F_st_* between geographically defined *Narnaviridae* populations produced an average *F_st_* of 0.046 and a maximum *F_st_* of 0.089, indicative of far less differentiation between geographically distinct populations of *Narnaviridae* than between individual populations (Fig. 7). Further, we found that geographic distance contributes significantly to observed genetic differentiation between these populations. A Mantel test using Spearman’s rank correlation, including Haversine distance and values of pairwise *F_st_*, was indicative of a significant correlation between geographic distance and genetic differentiation (*r* = 0.5988 *P* = .025). Further generating a scatterplot of geographic distances between any two populations showed a continuous cline of *F_st_*. These results suggest that as distance increases between any two geographically defined populations of hosts, there is an increase in the genetic differentiation between populations of associated *Narnaviridae* (Fig. 7).

### Host species best explains virome composition and diversity

Except in the case of *L. leucozonium*, no two halictid species of bee hosts shared the same virus species or family. Indeed, virus families were species-specific except for *Virgaviridae*, which includes mostly plant-pathogenic viral species. We then analyzed the viral diversity and richness of individual bee hosts. We first tested for differences in the number and diversity of virus OTUs (species and families) associated with the behaviors of halictid bees. When comparing all halictid bees sampled from the same locality (Princeton), we found no difference in the virus Richness or Shannon and Simpson indices (Richness: *P* = .585, Shannon: *P* = .184, Simpson: *P* = .156) (Supplementary Figs S1 and S2). We also compared differences in diversity of virus communities by including *A. mellifera* (also sampled from the same locality) and *L. leucozonium* (which was geographically dissimilar), but found no differences in effect (Richness: *P* = .5, Shannon: *P* = .201, Simpson: *P* = .195). We then investigated the effect of species association on virome richness and diversity. As above, we analyzed only the halictid hosts sampled from the same locality. Analysis via ANOVA and post hoc analysis with Tukey’s Honest Significance Test found no significant difference in Richness or Shannon and Simpson indices (Richness: *P* = .265, Shannon: *P* = .243, Simpson: *P* = .207) (Supplementary Figs S3 and S4). Though we found no effect of host sociality on the richness or diversity of host-associated RNA viral communities, our results are limited by the small sample size associated with each halictid species that was sampled from the Princeton locality.

**Figure 8:**
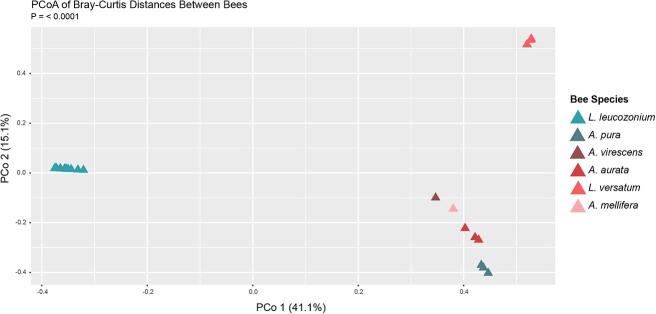
Bray–Curtis dissimilarity principal coordinate analysis based on absolute abundance of normalized transcript for each virus species OTU described in this study; each triangle represents an individual bee sample; PERMANOVA revealed that overall virus species composition was significantly distinguished by host species (*P* = .00001).

Despite no significant difference in virus species diversity and richness between different sympatric species of bee host, our sampling showed that distinct virus families were present in each of the different bee species and that species varied significantly in their virus species composition (Fig. 2A and B). Principal coordinate analysis using Bray–Curtis dissimilarity of the relative abundances of associated virus OTUs distributed the viromes into distinct clusters at both the species and family level (Adonis results: DF = 5, *R*^2^ = 0.824, *P* = .00001) (Fig. 8). Bees sampled from the same locality clustered closer together in ordinate space than with bees sampled from other localities, such as *L. leucozonium*), though virus OTUs from *L. versatum* (also sampled from the same locality) showed high dissimilarity. We note that the evidence for species-specificity by RNA viruses in sympatric halictid hosts are derived from a single population and would benefit from a higher number of sampled individuals across temporal and spatial distance. Our findings, that much of the variation in microbial species-associations is driven by the species of bee host, are consistent with other studies investigating interactions between sociality and microbiome composition in bees [25, 26, 72–75].

## Discussion

Here, we have used comparative metatranscriptomics to investigate the viromes of six species of bee, including five native halictid bees, across a spectrum of host sociality. Our sampling includes five wild bee species, drawn from both sympatric and allopatric populations, and our analysis identified six nearly complete genomes from 26 putatively novel virus species, derived from RdRP phylogeny, spanning across 12 virus families. The majority of the novel virus species fell into the family *Virgaviridae*, underlining the importance of pollinators as vectors of pollen-born plant pathogenic viruses. Virus species from the family *Iflaviridae*, containing some of the most virulent and pathogenic viruses affecting honey bees, appeared in samples of both honey bee and the wild *A. pura*. Though we observed the presence of deformed wing virus and Israeli acute paralysis virus in sampled honey bee hosts, we found no evidence for known honey bee virus in wild *A. pura* hosts despite sympatry. Future work examining opportunities for direct viral transmission between *A. mellifera* and other wild bee species would benefit from denser sampling of bee hosts, knowledge of the foraging frequency and specificity of wild bee species, and the frequency and magnitude of cross-species interactions. The potential of honey bee viruses, often associated with colony loss, to spill over into wild bee populations has important ecological implications as infected apiaries may serve as reservoirs for virus transmission. Overall, RNA viral transcripts detected in wild bees were relatively low compared to RNA viral transcripts found in *A. mellifera* and may reflect a healthy equilibrium between virus and host. This corroborates other studies investigating viral load in wild bee species. Dolezal *et al*. quantified the viral load of known honey bee viruses in 284 bees, including 124 Halicitidae, using RT-qPCR [6]. Honey bee viruses found in wild bees were found to be two-to-three orders of magnitude lower load than viruses in honey bees. Observations from both of these studies suggest that viral loads in wild bees are usually detected at low levels. It is important to note that it is not possible from this data to infer that low viral loads equate with low virulence in these hosts.

Though much is known about RNA viral ecology social bees due to studies in some domesticated bee species, such as *A. mellifera*, we sought to document the diversity, abundance, and structure of RNA viral communities associated with wild pollinators. As part of our sampling, we captured host halictid species along a gradient of sociality. Comparing populations of social bees to solitary bees, recent studies [25, 26, 73] have found little evidence for the influence of sociality on associated bacterial communities in wild, noncorbiculate bees. Though solitary bees in our study were associated with an absolute larger number of virus species, comparisons to closely related and sympatric social species revealed no effect of sociality on virus diversity or abundance though the limited number of samples collected for each halictid species in our study prevents drawing strong, general conclusions about the relationship between sociality and viral diversity in halictid bees. As the absence of evidence is not the evidence of absence, further work would incorporate both a deeper and wider sampling depth in natural halictid populations to account for the diversity of intra-host viral populations. Shell and Rehan [26] did find significant differences when examining the effect of sociality on intrapopulation variation in host-associated bacteria, and it is likely that our approach to RNA virus taxonomy (e.g. assigning species at 95% AAI) is too broad. Similar to the studies above, viral species and even families tended to be restricted to a single halictid species in our sampled hosts (Fig. 8). Despite similar ecology and nesting strategies, sympatric populations of four halictid host species were associated with distinct RNA viral species, suggesting some amount of host specificity in these viral species. In light of this association, we highlight the need for greater sampling depth, as our sample cohort represents a single population of only four wild bee species with four sampled *A. pura* individuals, four sampled *A. aurata* individuals, six sampled *L. versatum* individuals, and four sampled *A. virescens* individuals.

Further support for the specificity of viral association among these four halictid species would benefit from deeper and more uniform sampling across spatial and temporal distances and would be necessary to better understand the host range of the novel viral species described in this study. This is especially important as many of the plant pathogenic viral species described in this study are likely to be highly dissimilar from other populations of halictid hosts. Comparisons from this study against other populations of host-associated plant pathogenic viruses and putatively insect-associated viruses could help distinguish the association of further viral species among halictid hosts. Surprisingly, five allopatric populations of a single species, *L. leucozonium*, were all associated with the same species of *Narnaviridae*. It is unclear how *L. leucozonium* is associated with *Narnaviridae*, and it is surprising how little divergence has occurred despite the low replication fidelity of RNA viruses and over 600 km of distance between *L. leucozonium* population in this study. Though we note the absence of experiments validating *Narnaviridae* replication via negative-strand intermediates, *Narnaviridae* transcripts were on average 139× higher than plant pathogenic viral transcripts, and the consistency of a single *Narnaviridae* species across all 17 *L. leucozonium* warrants further investigation. Further work may investigate the presence of an endogenous retrovirus associated with *L. leucozonium* and the potential transcription of the retrovirus-associated loci.

The presence of *Narnaviridae* in all isolates of *L. leucozonium* allowed us to investigate the amount of virus diversity associated with allopatric populations of *L. leucozonium*. Two studies [26, 73] found that the microbiome of select wild bees species is influenced by its local environment. We investigated this and found significant evidence for isolation by distance in *Narnaviridae* metapopulations. Though individual pairwise comparisons of *Narnaviridae* population diversity found no association with Haversine distance, these findings suggest that *Narnaviridae* populations associated with individual *L. leucozonium* hosts found within the same geographic locality are as different from one another as they are from sampled hosts more than 600 km away. This high population differentiation can be explained by the low fidelity rates shared by most RNA viruses [9] and by little to no migration between individual virus populations. Additionally, these virus populations may be a result of recent infection, and the high amount of genetic diversity within these populations may reflect a history of recent population expansion. Treating all *Narnaviridae* populations within a single sampling site as a metapopulation revealed a significant correlation between genetic and Haversine distance. This association may reflect signatures of host–virus coevolution as higher relatedness between sympatric populations of *L. leucozonium* might increase the frequency of alleles of specific immune genes and lead to less varied virus populations. Further work comparing loci associated with known immune genes in geographically distinct hosts might provide support for this hypothesis.

Given the importance of RNA viruses to pollinator health, it is critical that we understand the interactions between wild bee species and their virus symbionts. Though virus research in the honey bee system is undoubtedly important and has taught us much about Hymenoptera–virus interactions, it is becoming increasingly clear that interactions and potential mechanisms between microorganisms and their bee hosts are species-specific. However, it is surprising to note the consistency of this specificity regardless of host behaviors or geography. Eusocial organisms have long been a fascination and special difficulty of evolutionary biology [76], and the relatively simple viromes of this socially plastic family of bees, in addition to our understanding of the honey bee metagenome, incites further discovery, especially in the context of viral discovery and viral ecology.

## Supplementary Material

921_1_supp_32726_s0zvlz_convrt_ycad003

SupplementalTable1_ycad003

SupplementalTable2_ycad003

SupplementalTable3_ycad003

## Data Availability

Metagenomic datasets for each bee sample have been deposited into the NCBI SRA database. The accession numbers for these samples are SAMN33439837–SAMN33439874 under the BioProject PRJNA938525. GenBank accessions number for viral contigs used in this study can are OR533014 - OR533099. Scripts for the ${F}_{st}$ calculations are available on GitHub.
