# Supplementary material for: Host species and geography impact bee-associated RNA virus communities with evidence for isolation by distance in viral populations
Source: ISME Commun. 2024 Jan 10;4(1):ycad003. doi: 10.1093/ismeco/ycad003 (PMC10833078; doi:10.1093/ismeco/ycad003)
Supplement: 921_1_supp_32726_s0zvlz_convrt_ycad003 [file 921_1_supp_32726_s0zvlz_convrt_ycad003.pdf]

1 Supplemental Figures

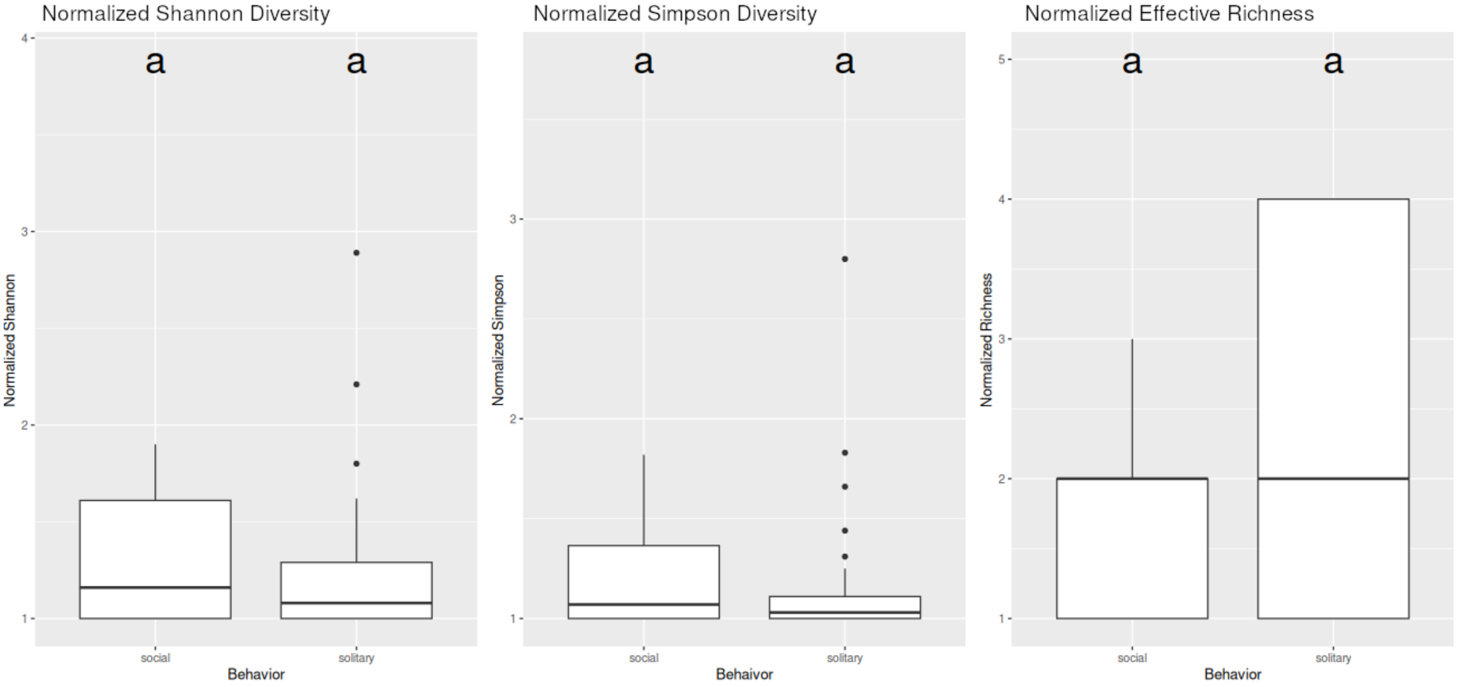

Figure 1: Alpha diversity metrics (Shannon index, Simpson index, and Richness) describing differences in viral species diversity associated with social and solitary bees.

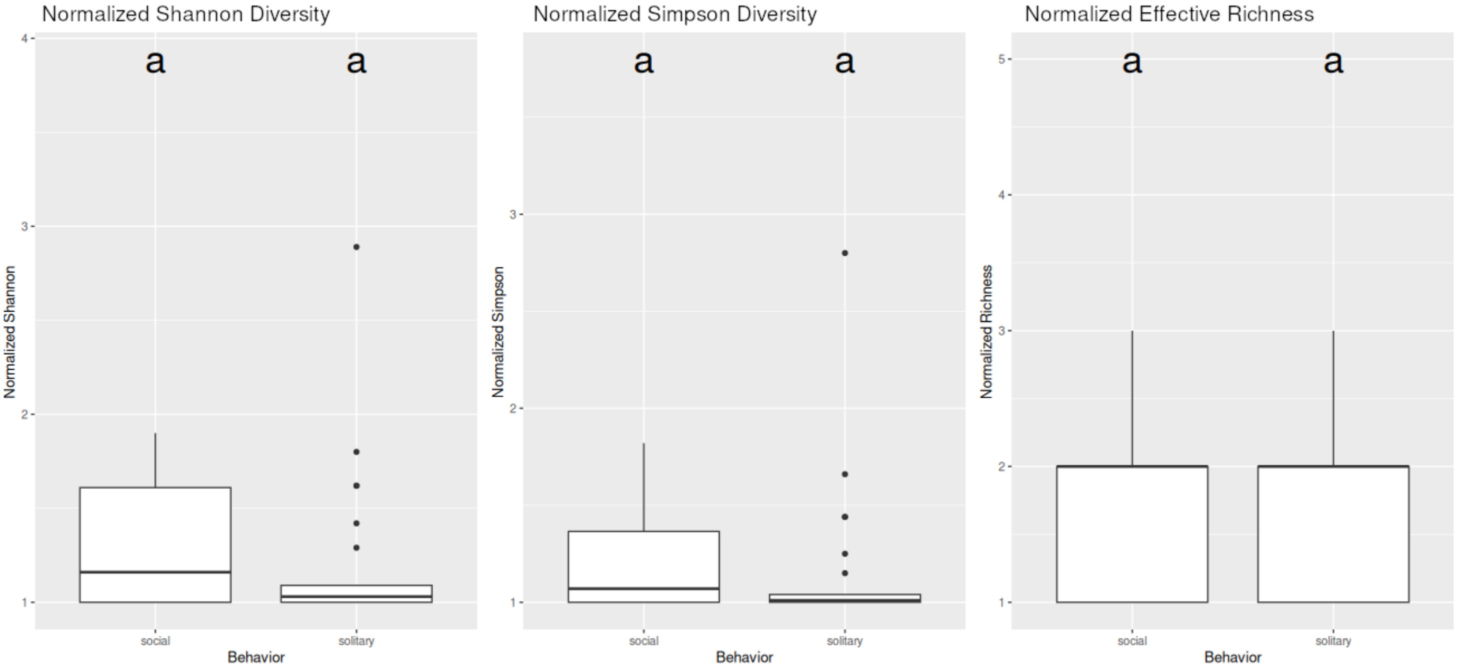

Figure 2: Alpha diversity metrics (Shannon index, Simpson index, and Richness) describing differences in viral family diversity associated with social and solitary bees. Letters correspond to groupings by Tukey's Honest Significant Difference Test

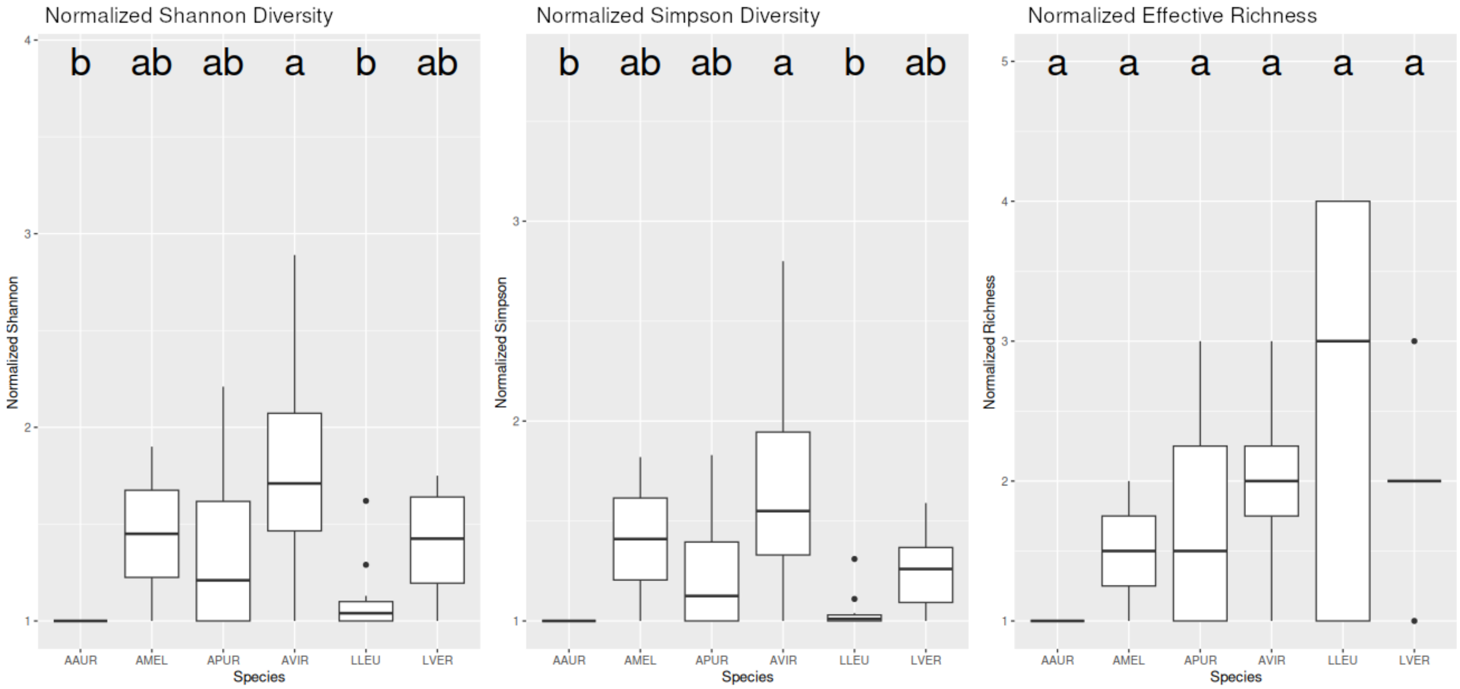

Figure 3: Alpha diversity metrics (Shannon index, Simpson index, and Richness) describing differences in viral species diversity associated with different bee species. Letters correspond to groupings by Tukey's Honest Significant Difference Test

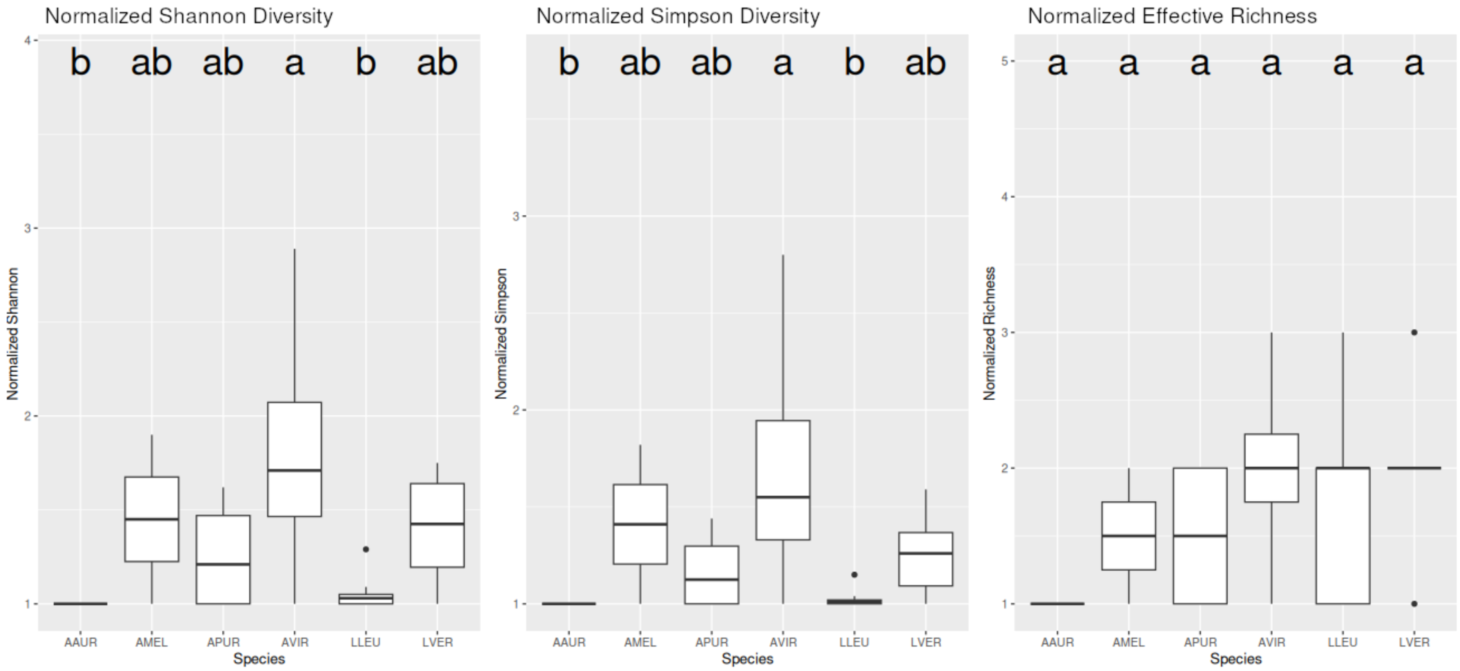

Figure 4: Alpha diversity metrics (Shannon index, Simpson index, and Richness) describing differences in viral family diversity associated with different bee species. Letters correspond to groupings by Tukey's Honest Significant Difference Test.

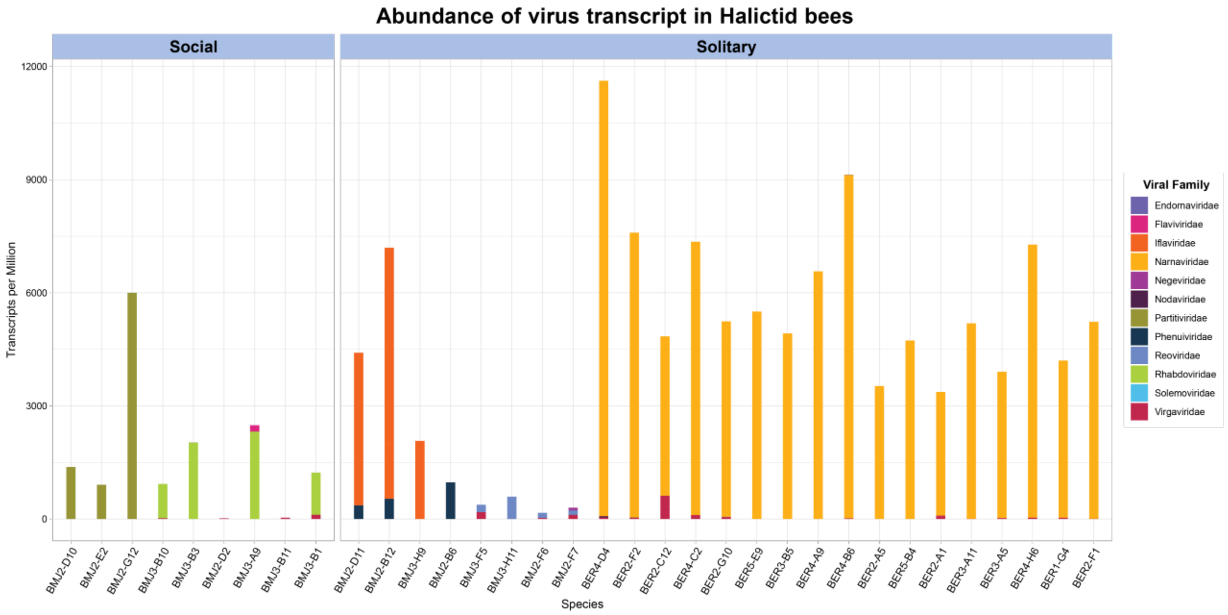

Figure 5: Absolute abundance of normalized RdRP virus transcripts across sampled halictid bees in this study. Each bar plot along the X-axis represents an individual sampled bee and the Y-axis represents absolute number of virus reads assigned to each family. Colors within each bar represent reads assigned to a given virus family.

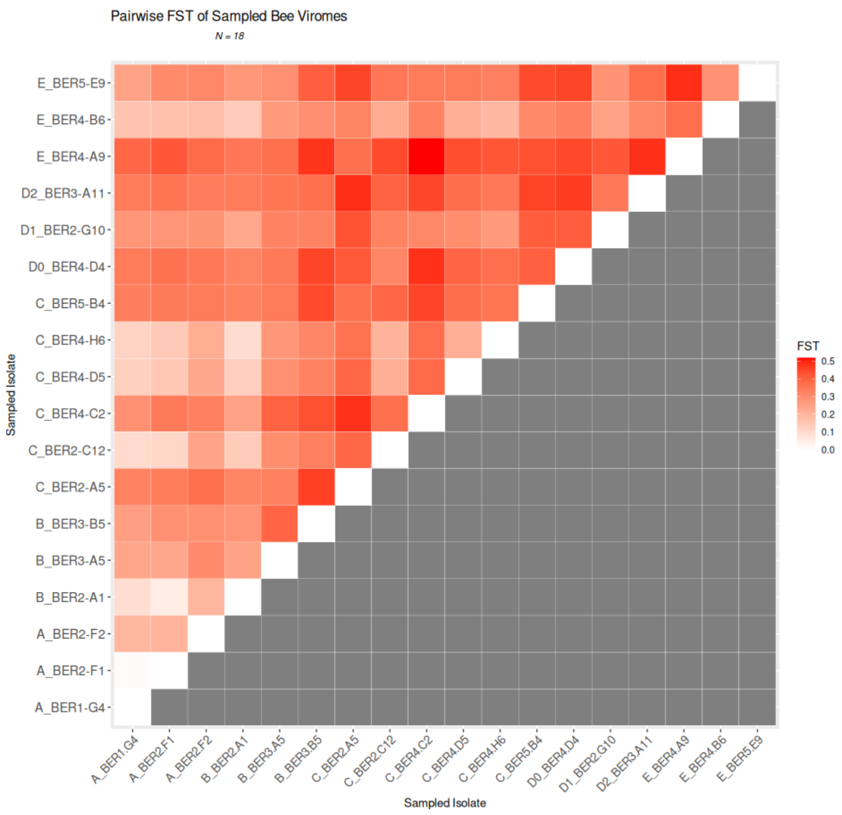

Figure 6: A matrix of pairwise F_{st} comparisons representing patterns of genetic differentiation among 17 individual populations of *Narnaviridae* viromes. Color represents the degree of genetic differentiation between any pair of combinations. Darker color indicates higher values of differentiation. Genetic differentiation is defined by a scale on the right side of the figure.

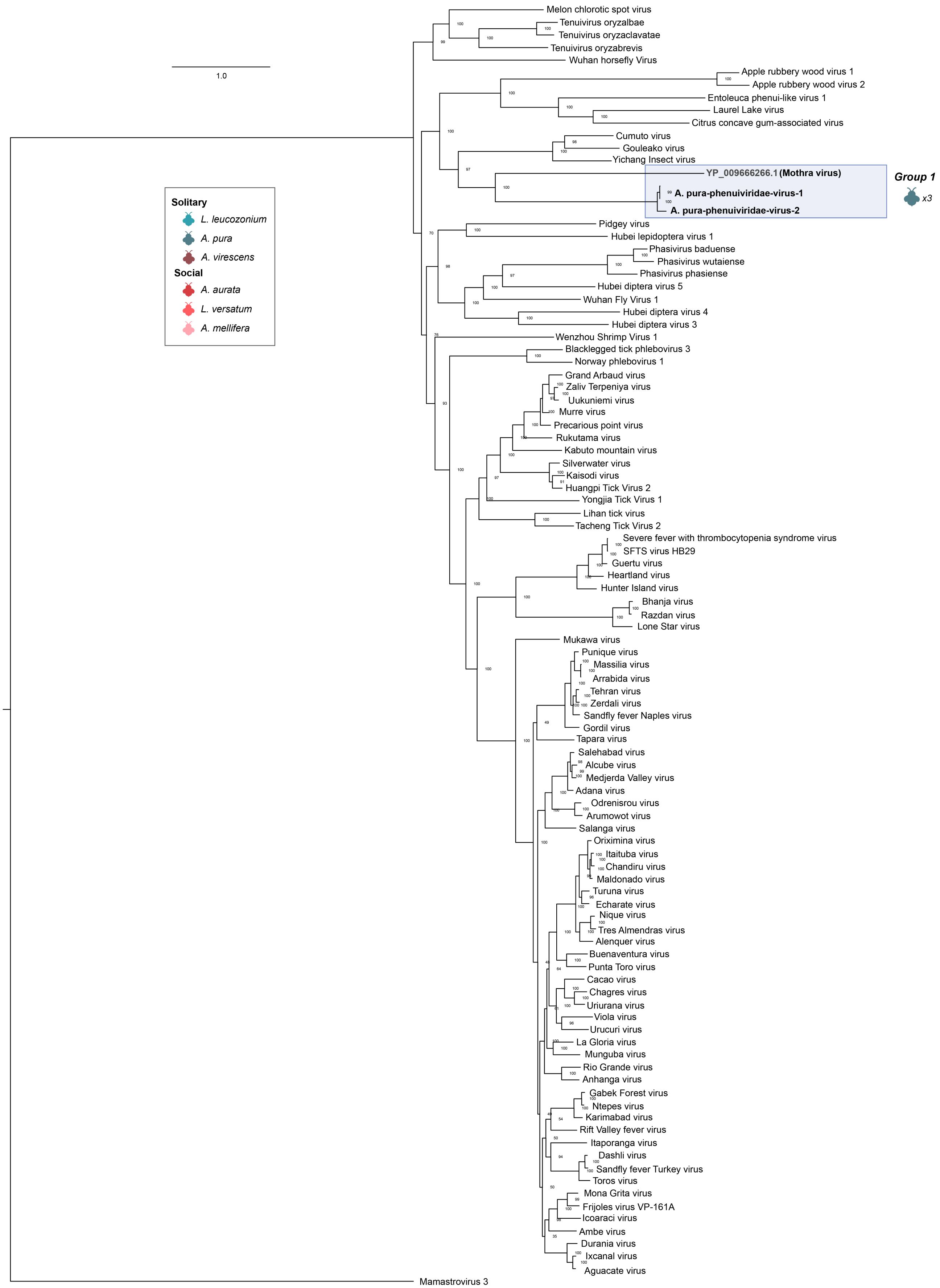

Figure 7: RdRP phylogeny of *Phenuiviridae* viruses. Tree is midpoint rooted for clarity only. Viruses sampled in this study are detailed highlighted in blue. Bootstrap values for select nodes are provided. Bee icon color represent species host that viral group was associated with; number represent the number of bee samples associated with viral group. The scale bar represents the number of amino acid substitutions.

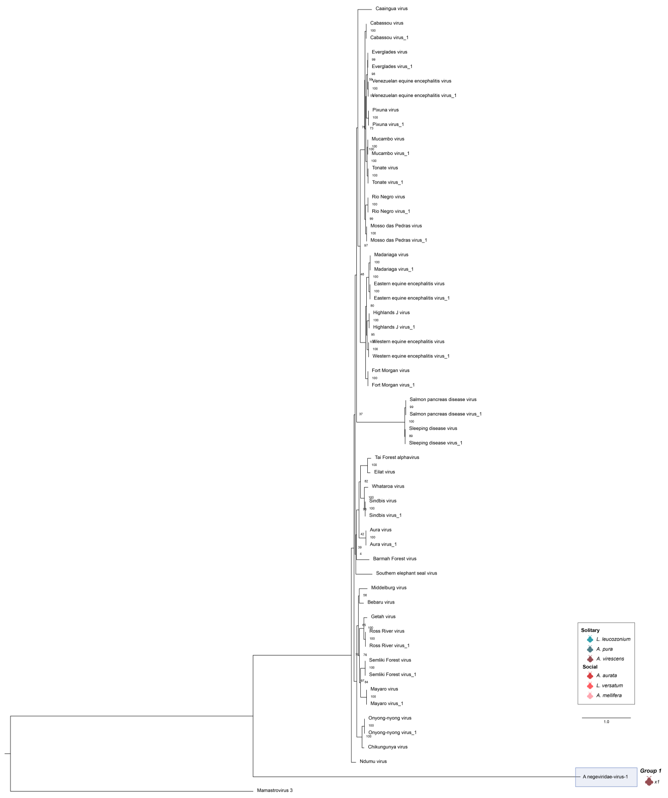

Figure 8: RdRP phylogeny of *Negeviridae* viruses. Tree is midpoint rooted for clarity only. Viruses sampled in this study are detailed highlighted in blue. Bootstrap values for select nodes are provided. Bee icon color represent species host that viral group was associated with; number represent the number of bee samples associated with viral group. The scale bar represents the number of amino acid substitutions.

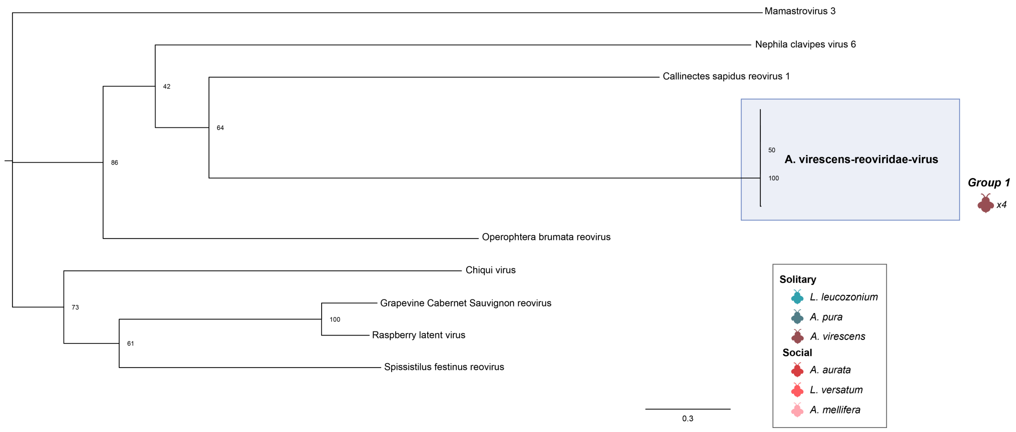

Figure 9: RdRP phylogeny of *Reoviridae* viruses. Tree is midpoint rooted for clarity only. Viruses sampled in this study are detailed highlighted in blue. Bootstrap values for select nodes are provided. Bee icon color represent species host that viral group was associated with; number represent the number of bee samples associated with viral group. The scale bar represents the number of amino acid substitutions.

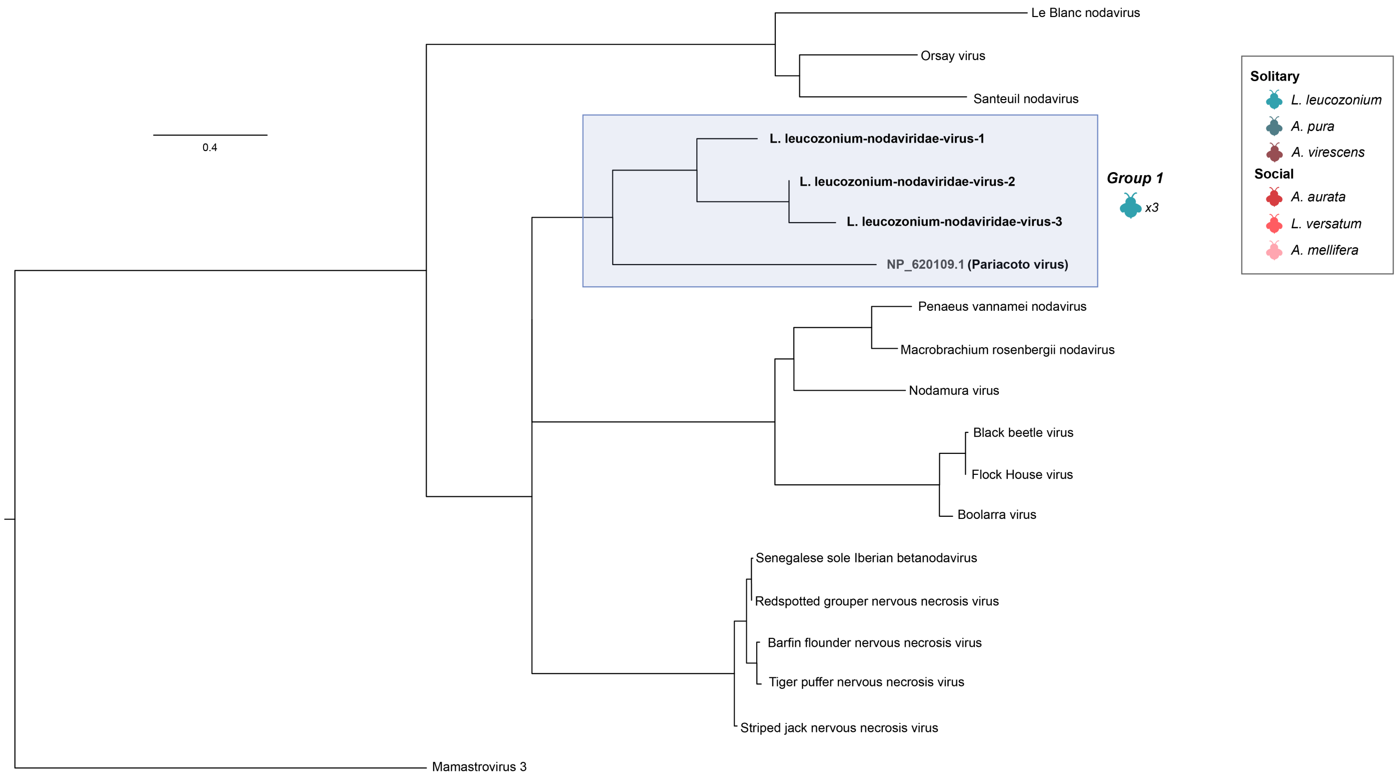

Figure 10: RdRP phylogeny of *Nodaviridae* viruses. Tree is midpoint rooted for clarity only. Viruses sampled in this study are detailed highlighted in blue. Bootstrap values for select nodes are provided. Bee icon color represent species host that viral group was associated with; number represent the number of bee samples associated with viral group. The scale bar represents the number of amino acid substitutions.

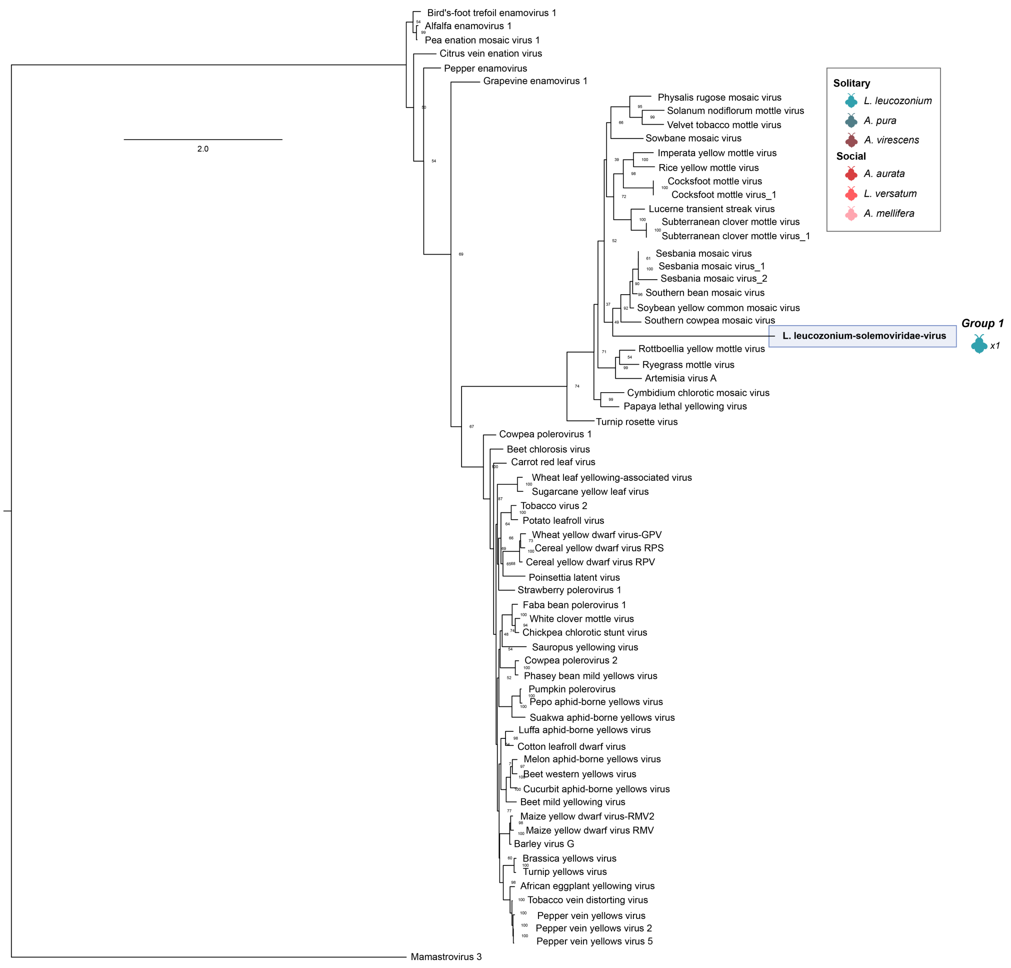

Figure 11: RdRP phylogeny of *Solemoviridae* viruses. Tree is midpoint rooted for clarity only. Viruses sampled in this study are detailed highlighted in blue. Bootstrap values for select nodes are provided. Bee icon color represent species host that viral group was associated with; number represent the number of bee samples associated with viral group. The scale bar represents the number of amino acid substitutions.

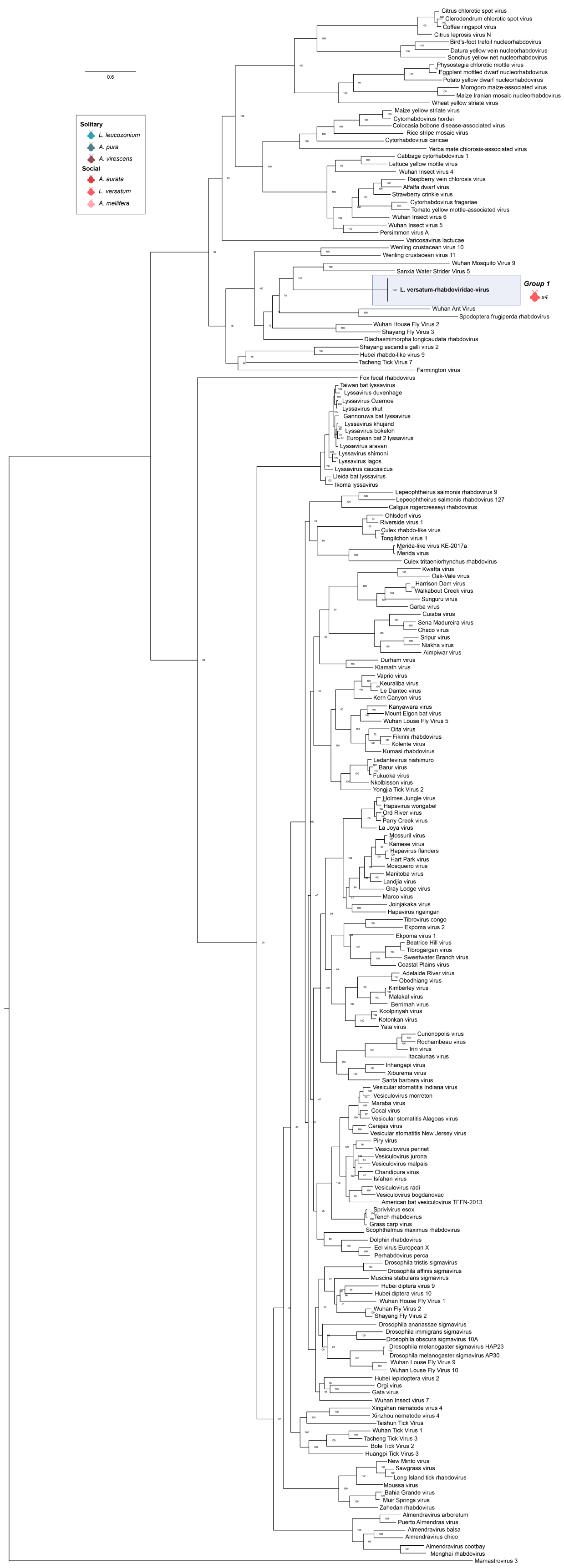

Figure 12: RdRP phylogeny of *Rhabdoviridae* viruses. Tree is midpoint rooted for clarity only. Viruses sampled in this study are detailed highlighted in blue. Bootstrap values for select nodes are provided. Bee icon color represent species host that viral group was associated with; number represent the number of bee samples associated with viral group. The scale bar represents the number of amino acid substitutions.

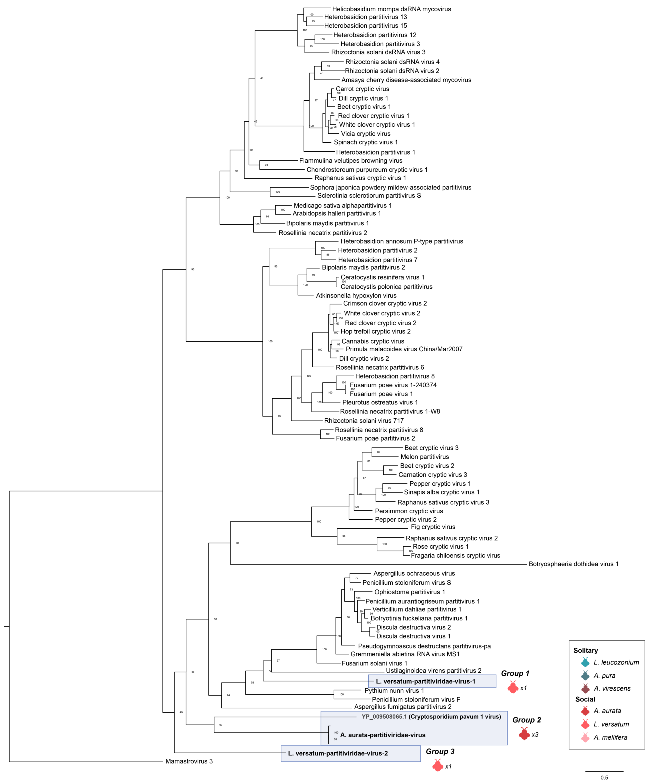

Figure 13: RdRP phylogeny of *Partitiviridae* viruses. Tree is midpoint rooted for clarity only. Viruses sampled in this study are detailed highlighted in blue. Bootstrap values for select nodes are provided. Bee icon color represent species host that viral group was associated with; number represent the number of bee samples associated with viral group. The scale bar represents the number of amino acid substitutions.

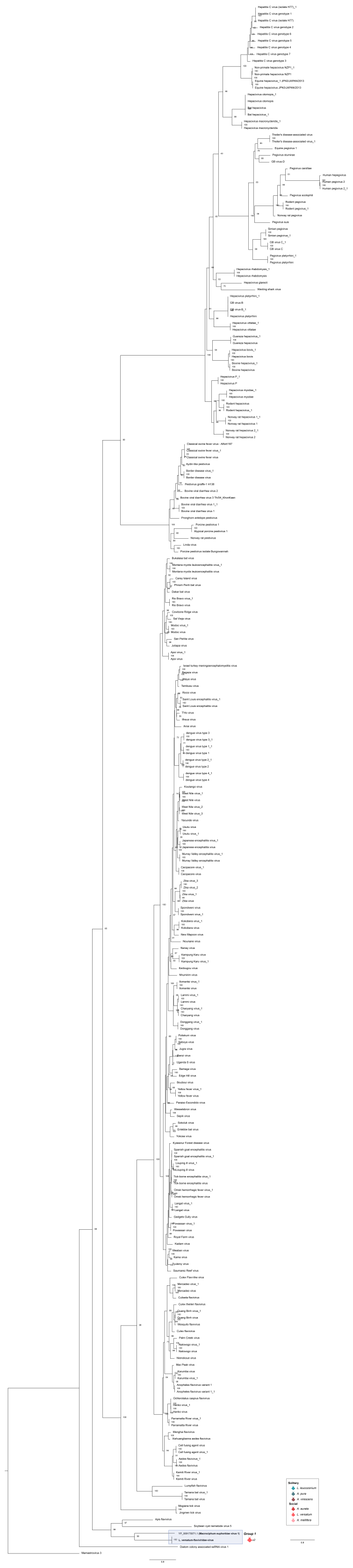

Figure 14: RdRP phylogeny of *Flaviviridae* viruses. Tree is midpoint rooted for clarity only. Viruses sampled in this study are detailed highlighted in blue. Bootstrap values for select nodes are provided. Bee icon color represent species host that viral group was associated with; number represent the number of bee samples associated with viral group. The scale bar represents the number of amino acid substitutions.

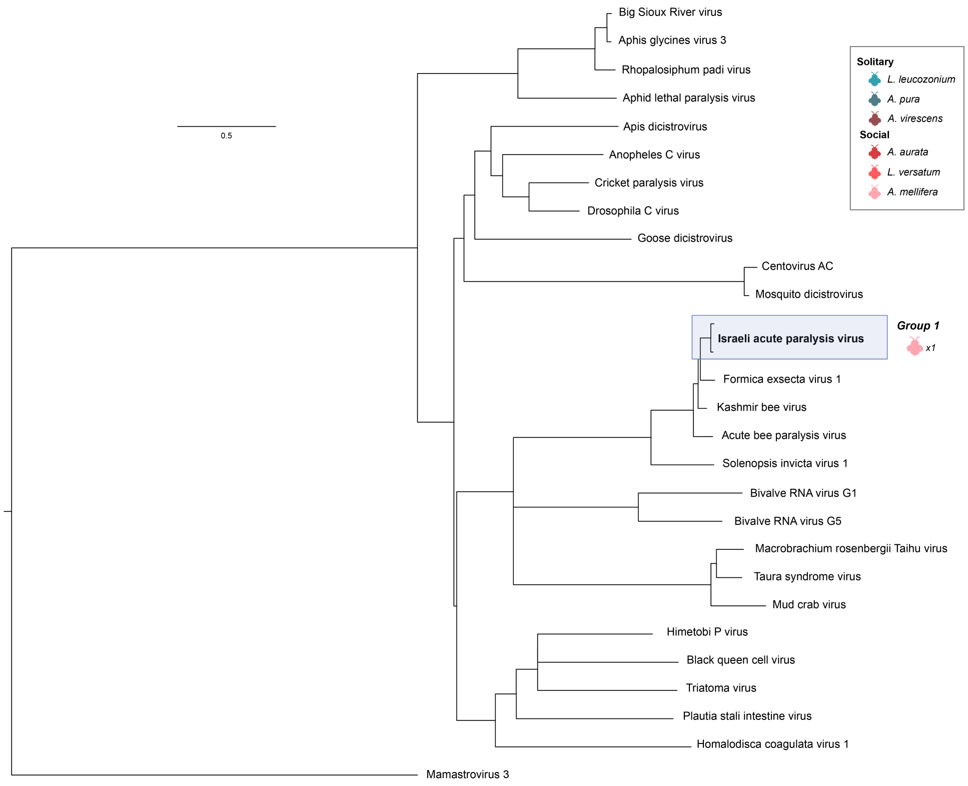

Figure 15: RdRP phylogeny of *Dicistroviridae* viruses. Tree is midpoint rooted for clarity only. Viruses sampled in this study are detailed highlighted in blue. Bootstrap values for select nodes are provided. Bee icon color represent species host that viral group was associated with; number represent the number of bee samples associated with viral group. The scale bar represents the number of amino acid substitutions.

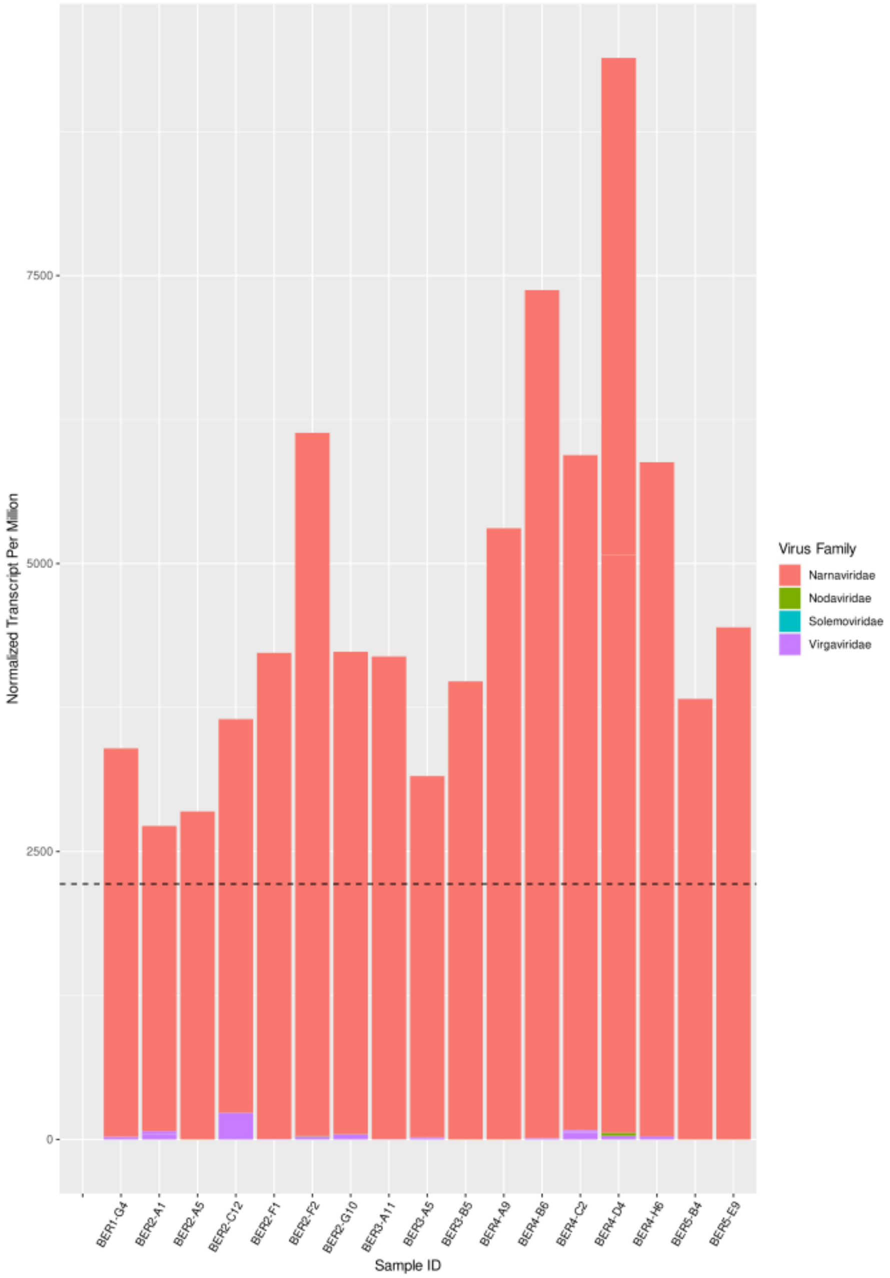

Figure 16: Absolute abundance of normalized RdRP virus transcripts across sampled *L. leucozonium* bee hosts in this study. Each bar plot along the X-axis represents an individual sampled bee and the Y-axis represents absolute number of virus reads assigned to each family. Colors within each bar represent reads assigned to a given virus family. A black dotted line indicates the average normalized viral transcript across all halictid bees sampled in this study.

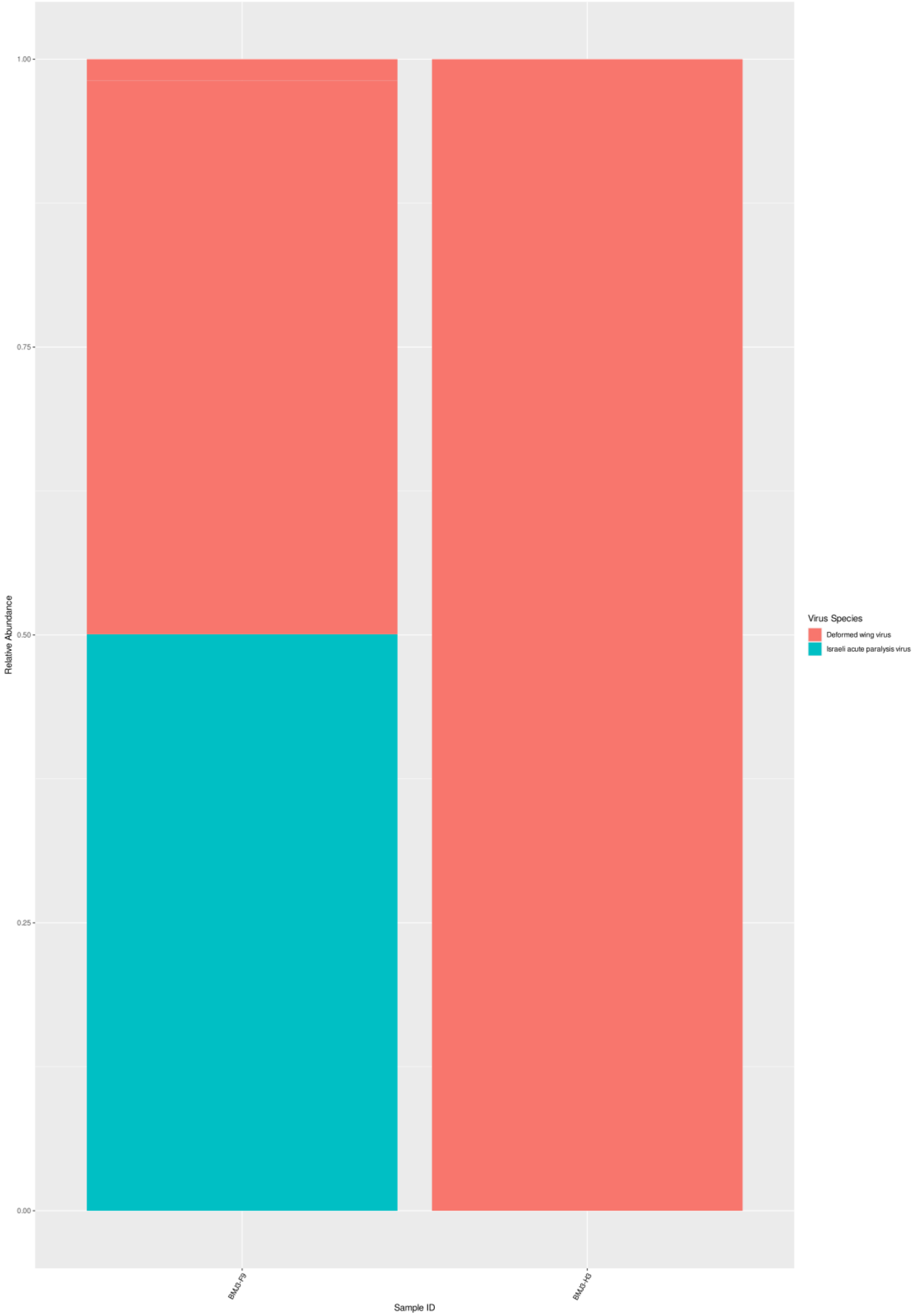

Figure 17: Relative abundance of normalized RdRP virus transcripts across sampled *A. mellifera*. Each bar plot along the X-axis represents an individual sampled bee and the Y-axis represents the relative abundance of reads assigned to each species. Colors within each bar represent reads assigned to a given virus species.

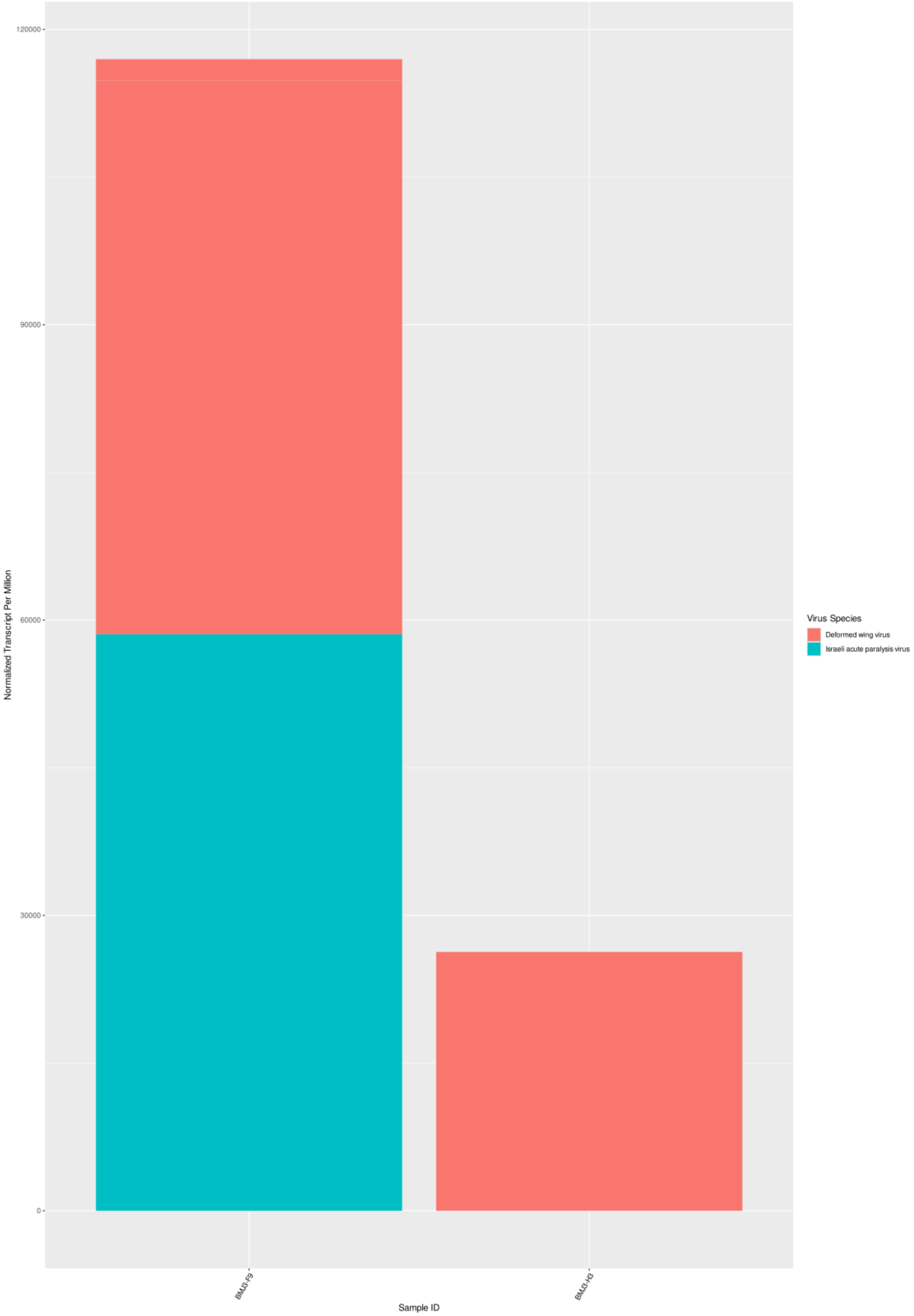

Figure 18: Absolute abundance of normalized RdRP virus transcripts across sampled *A. mellifera*. Each bar plot along the X-axis represents an individual sampled bee and the Y-axis represents absolute number of virus reads assigned to each species. Colors within each bar represent reads assigned to a given virus species.

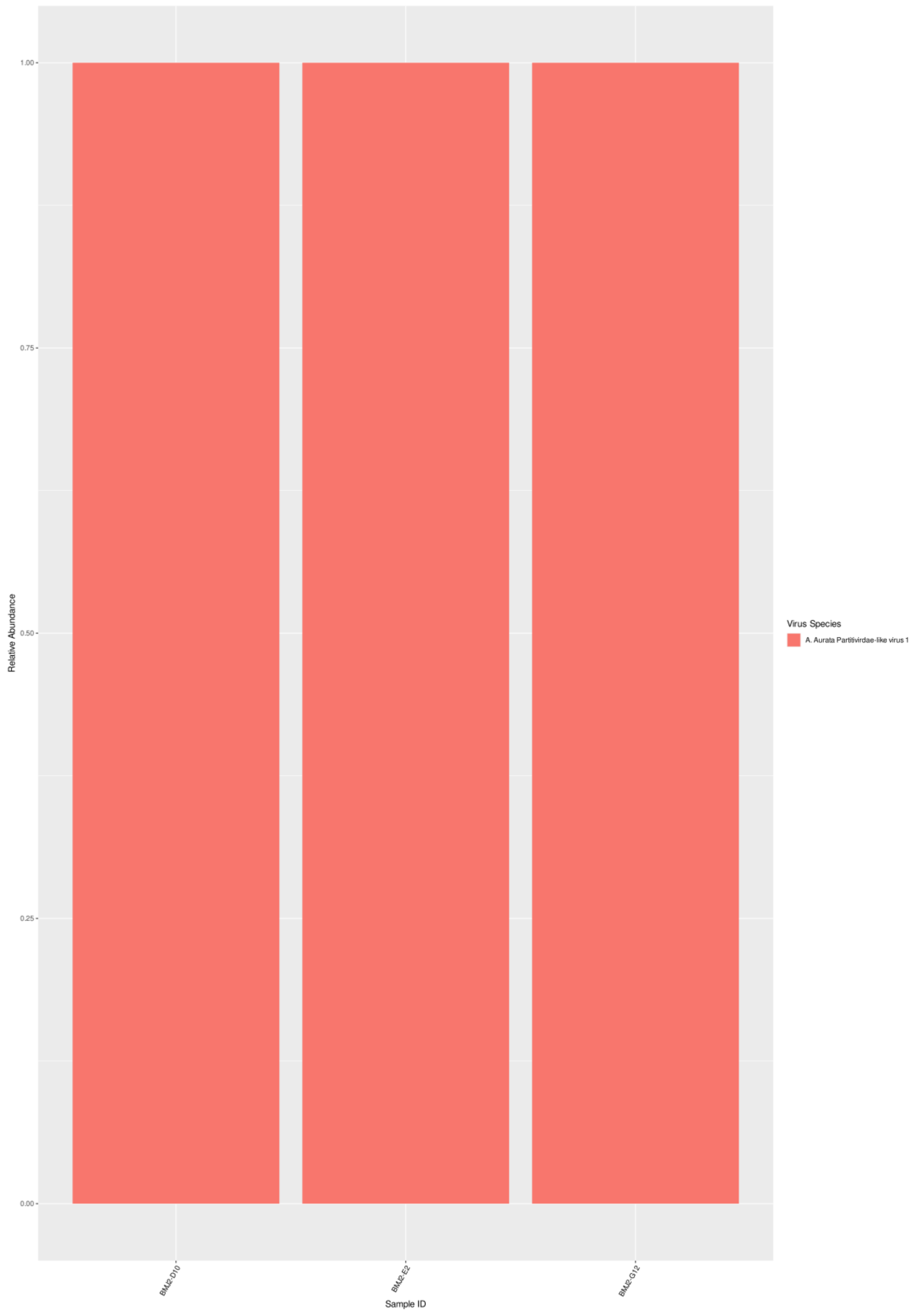

Figure 19: Relative abundance of normalized RdRP virus transcripts across sampled *A. aurata*. Each bar plot along the X-axis represents an individual sampled bee and the Y-axis represents the relative abundance of reads assigned to each species. Colors within each bar represent reads assigned to a given virus species.

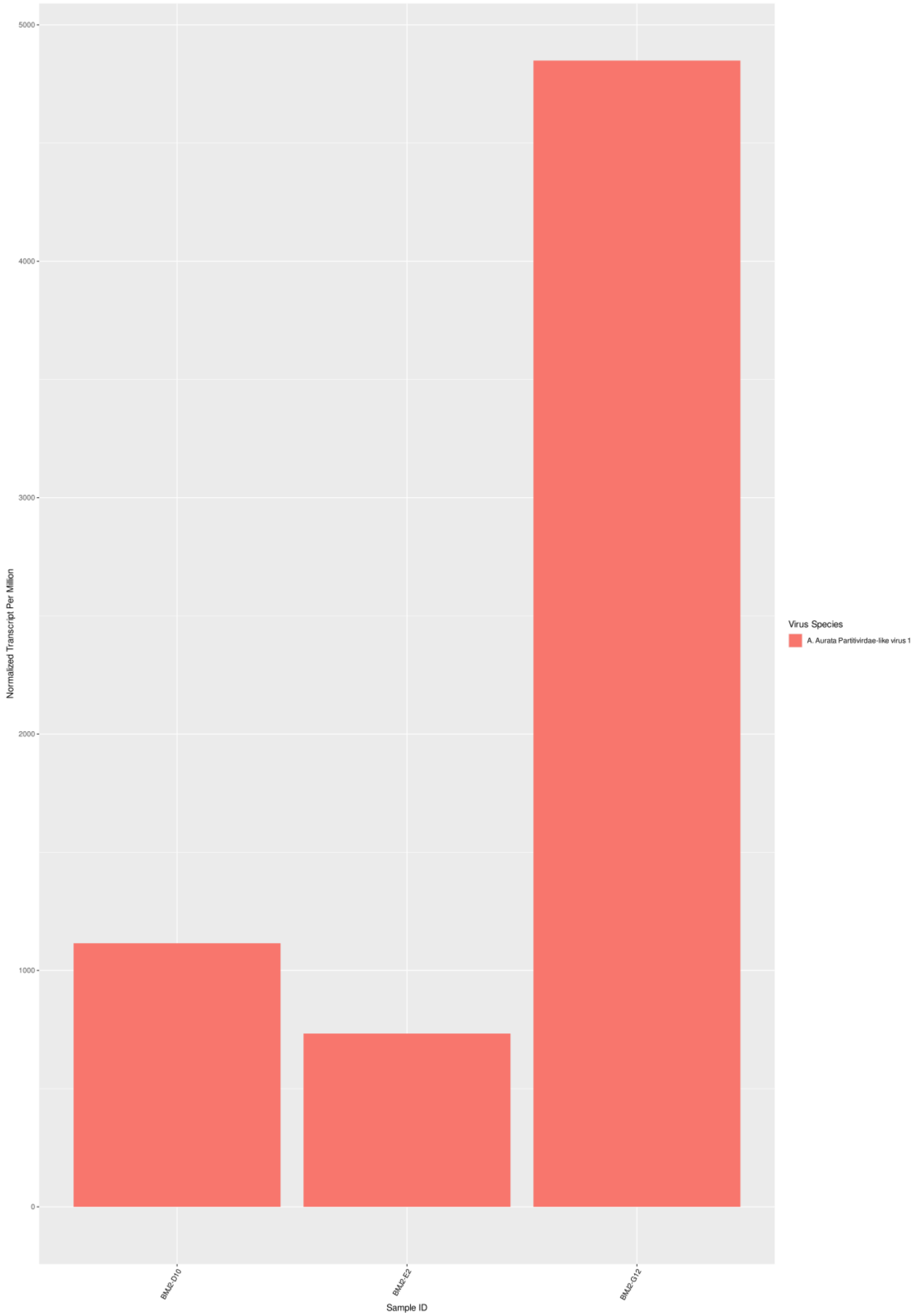

Figure 20: Absolute abundance of normalized RdRP virus transcripts across sampled *A. aurata*. Each bar plot along the X-axis represents an individual sampled bee and the Y-axis represents absolute number of virus reads assigned to each species. Colors within each bar represent reads assigned to a given virus species.

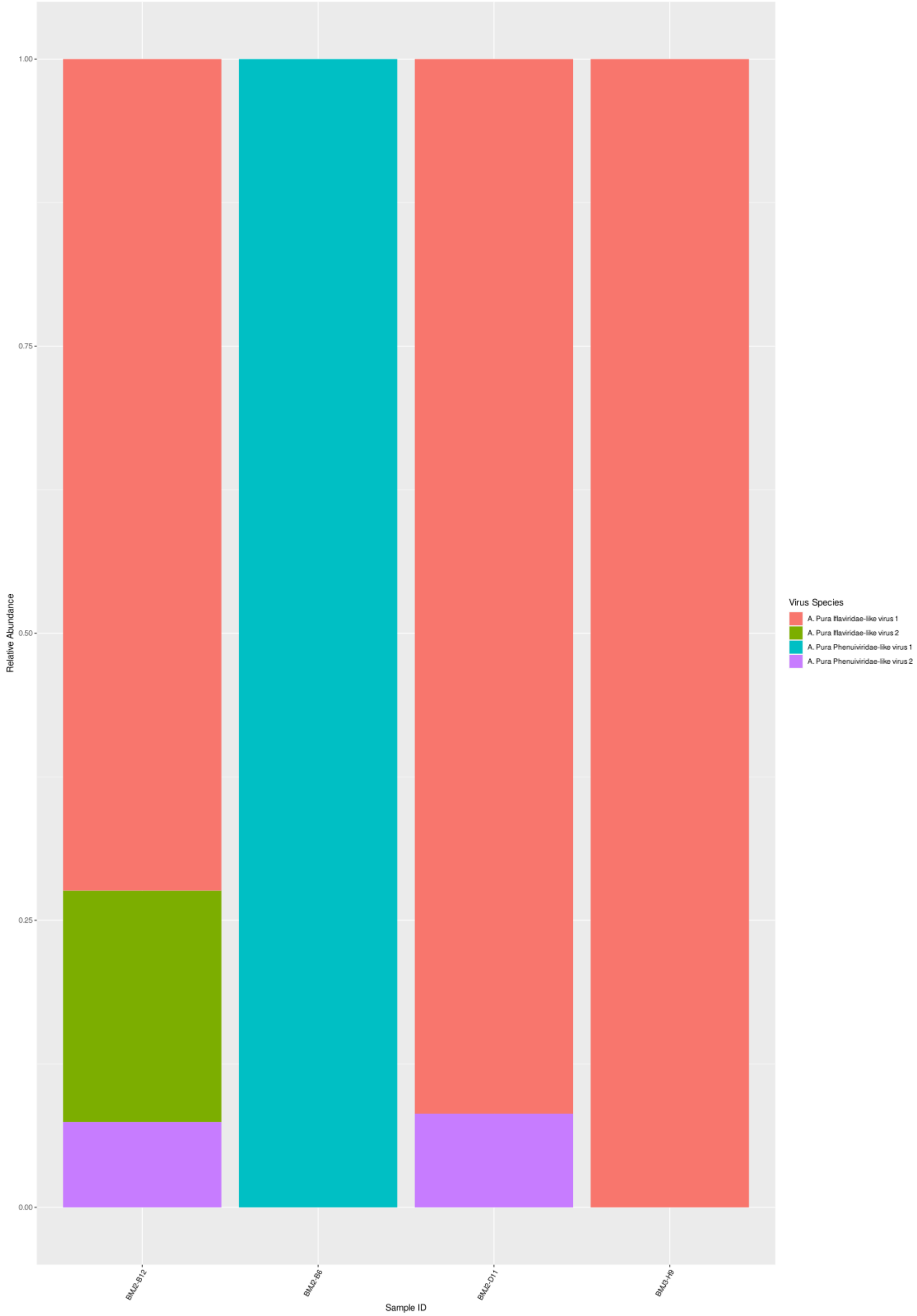

Figure 21: Relative abundance of normalized RdRP virus transcripts across sampled *A. pura*. Each bar plot along the X-axis represents an individual sampled bee and the Y-axis represents the relative abundance of reads assigned to each species. Colors within each bar represent reads assigned to a given virus species.

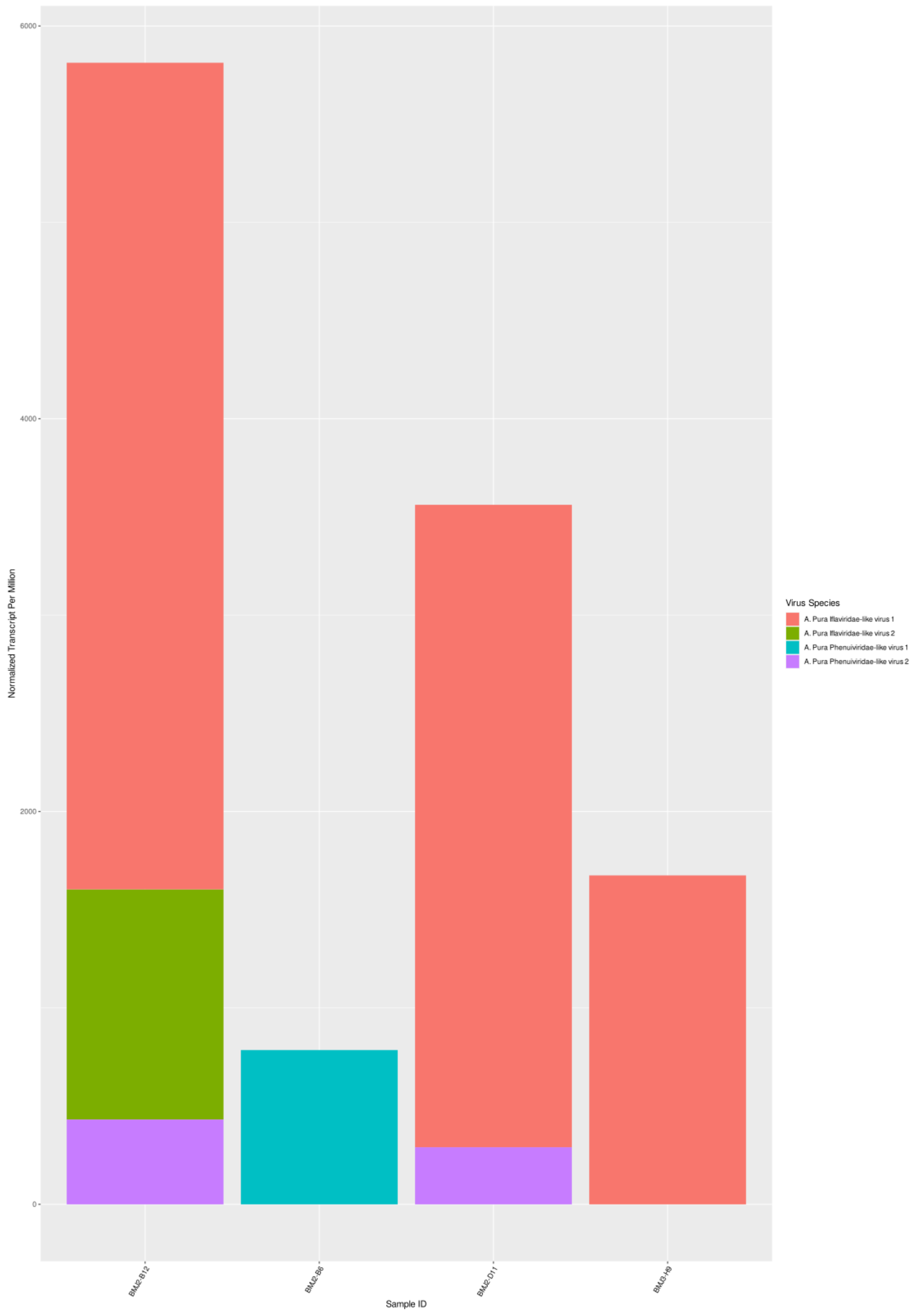

Figure 22: Absolute abundance of normalized RdRP virus transcripts across sampled *A. pura*. Each bar plot along the X-axis represents an individual sampled bee and the Y-axis represents absolute number of virus reads assigned to each species. Colors within each bar represent reads assigned to a given virus species.

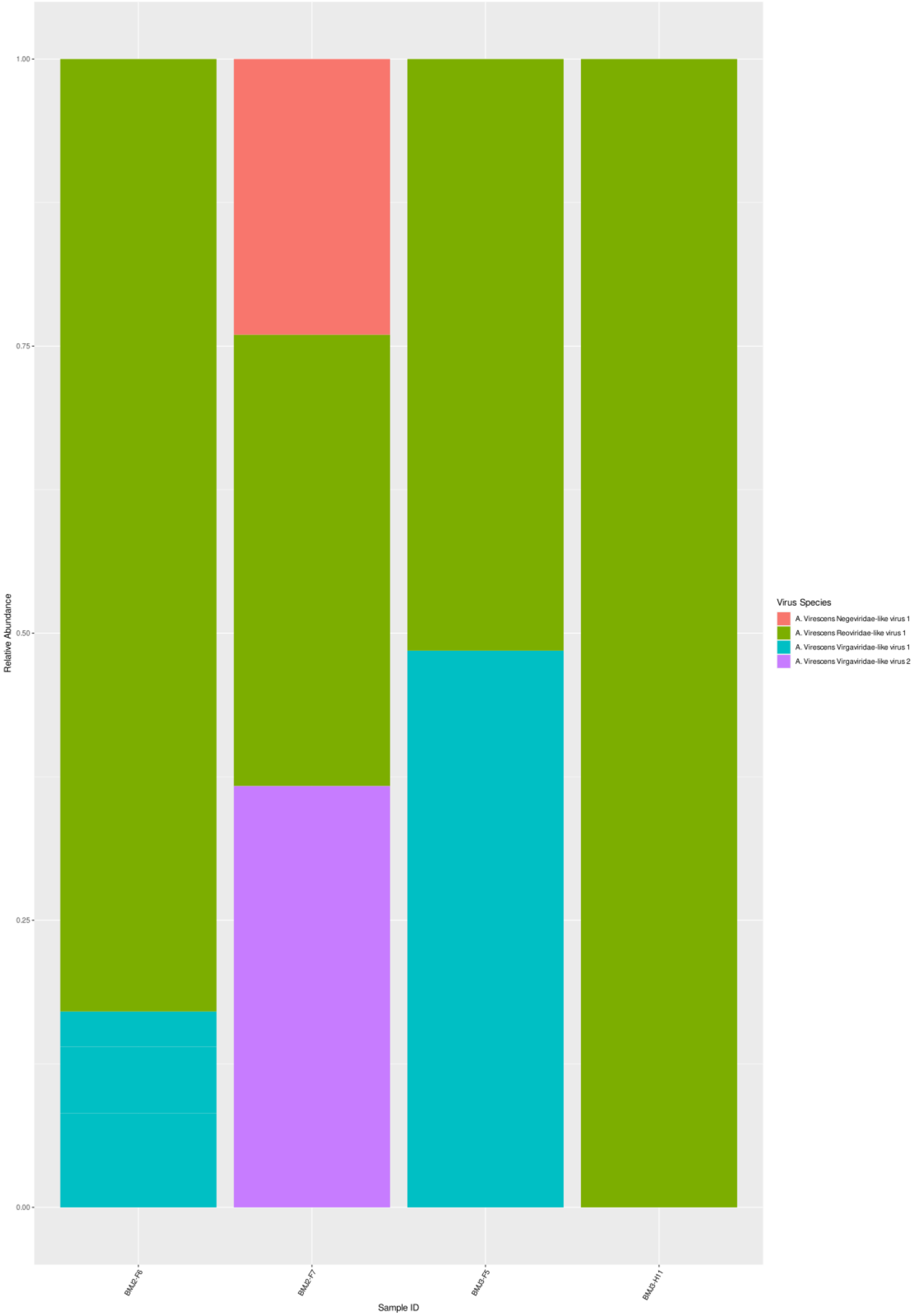

Figure 23: Relative abundance of normalized RdRP virus transcripts across sampled *A. virescens*. Each bar plot along the X-axis represents an individual sampled bee and the Y-axis represents the relative abundance of reads assigned to each species. Colors within each bar represent reads assigned to a given virus species.

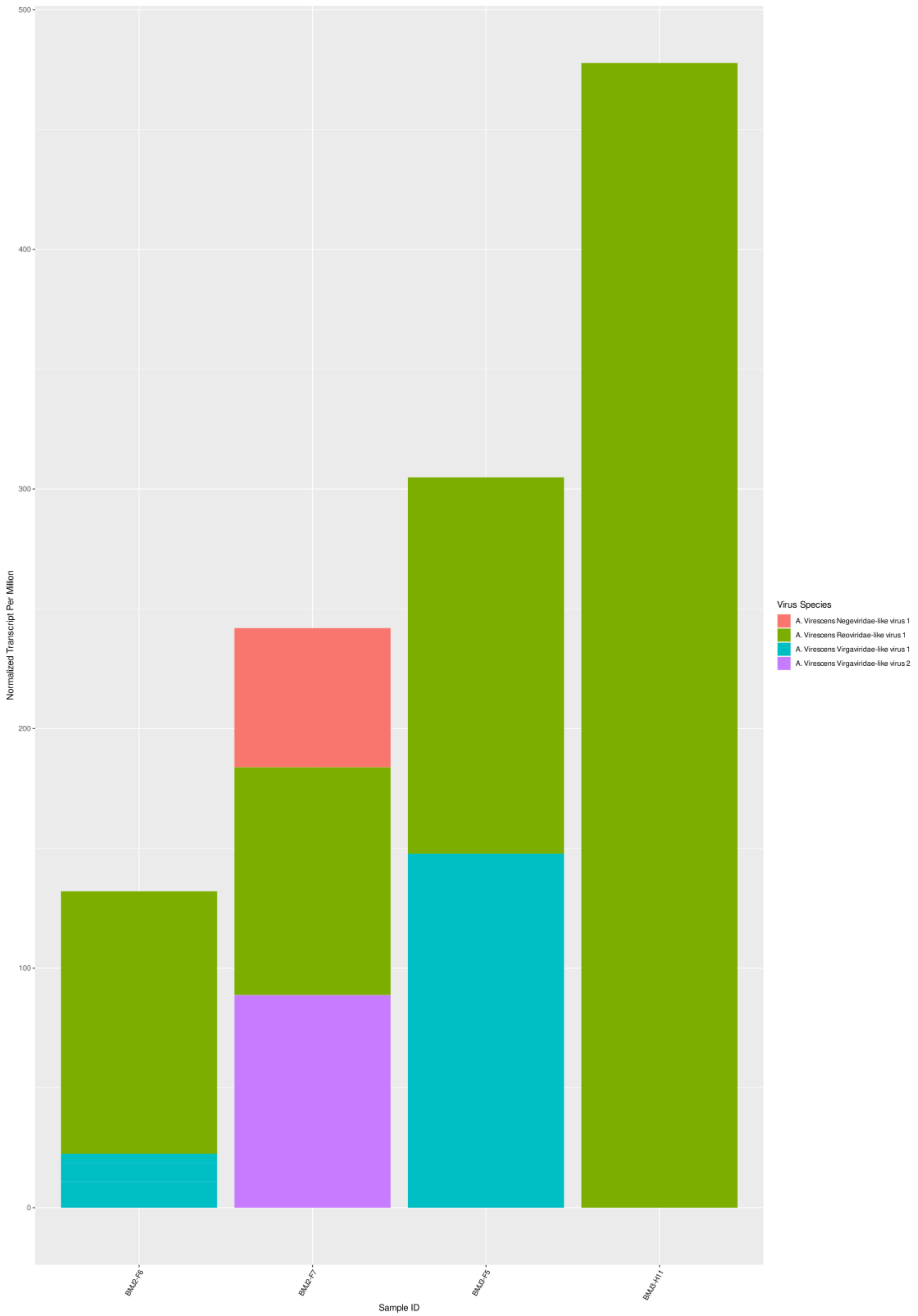

Figure 24: Absolute abundance of normalized RdRP virus transcripts across sampled *A. virescens*. Each bar plot along the X-axis represents an individual sampled bee and the Y-axis represents absolute number of virus reads assigned to each species. Colors within each bar represent reads assigned to a given virus species.

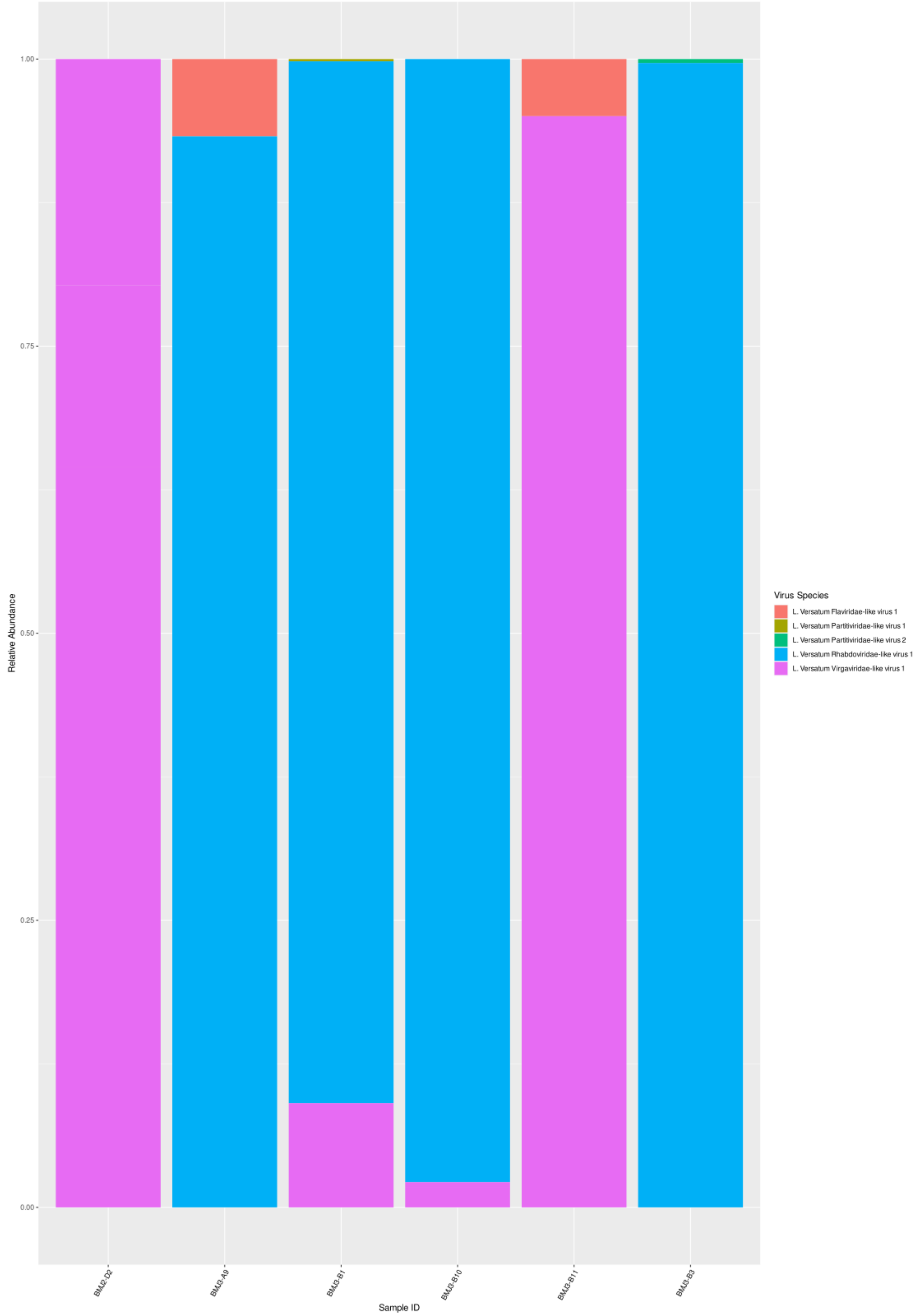

Figure 25: Relative abundance of normalized RdRP virus transcripts across sampled *L. versatum*. Each bar plot along the X-axis represents an individual sampled bee and the Y-axis represents the relative abundance of reads assigned to each species. Colors within each bar represent reads assigned to a given virus species.

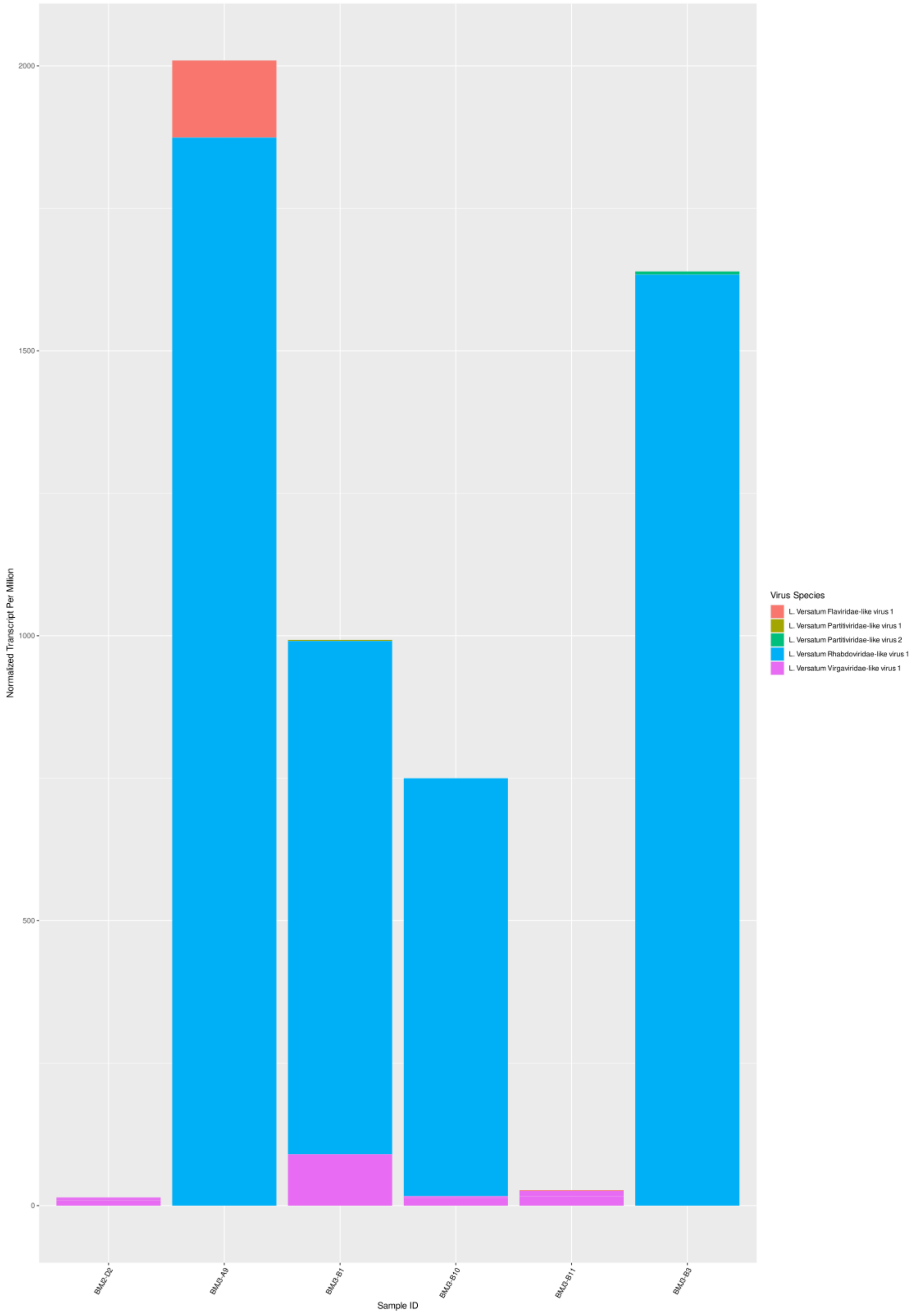

Figure 26: Absolute abundance of normalized RdRP virus transcripts across sampled *L. versatum*. Each bar plot along the X-axis represents an individual sampled bee and the Y-axis represents absolute number of virus reads assigned to each species. Colors within each bar represent reads assigned to a given virus species.

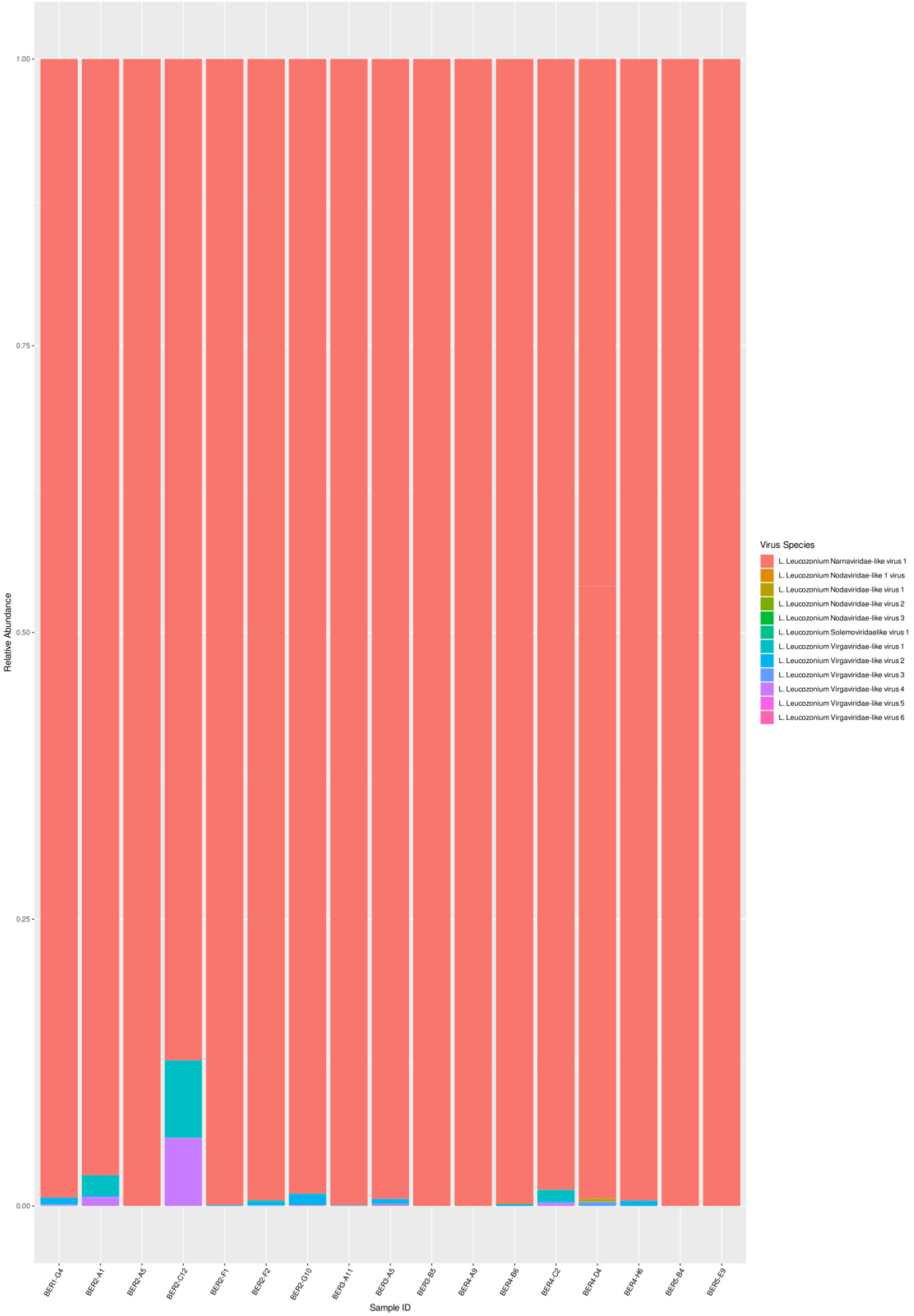

Figure 27: Relative abundance of normalized RdRP virus transcripts across sampled *L. leucozonium*. Each bar plot along the X-axis represents an individual sampled bee and the Y-axis represents the relative abundance of reads assigned to each species. Colors within each bar represent reads assigned to a given virus species.

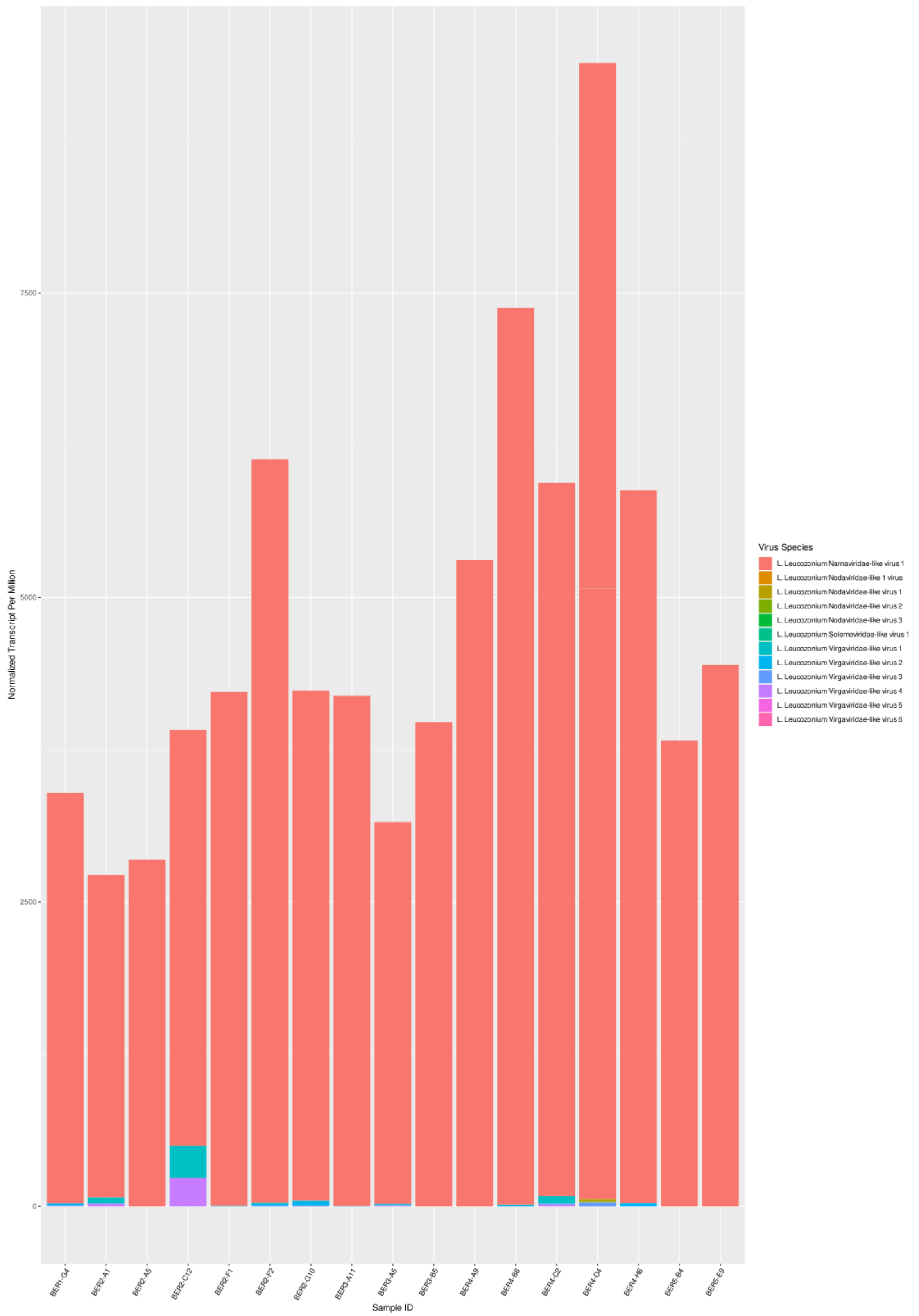

Figure 28: Absolute abundance of normalized RdRP virus transcripts across sampled *L. leucozonium*. Each bar plot along the X-axis represents an individual sampled bee and the Y-axis represents absolute number of virus reads assigned to each species. Colors within each bar represent reads assigned to a given virus species.
